# Generalized nonlinear weakly singular retarded integral inequalities with maxima and their applications

**DOI:** 10.1186/s13660-018-1885-6

**Published:** 2018-10-26

**Authors:** Yong Yan, Derong Zhou, Jianglin Zhao

**Affiliations:** 1 School of Science and Technology, Sichuan Minzu College, Kangding, P.R. China; 2 Network Information Center, Sichuan Minzu College, Kangding, P.R. China

**Keywords:** 26D15, 34A08, 34A34, Integral inequalities, Maxima, Monotonicity, Uniqueness

## Abstract

This paper deals with a generalized nonlinear weakly singular retarded Wendroff-type integral inequality with maxima of an unknown function of two variables. The key is that a technique of monotonization without separability and monotonicity of given functions is used for estimating the boundedness of unknown functions. Then our outcomes can be helpful to weaken conditions for some known results. By applying our results, the uniqueness of solutions for some singular integral equation with maxima may be proven.

## Introduction

The Gronwall inequality [1] holds a vital place in studying qualitative properties of the solutions of integral equations and differential equations. Some linear and nonlinear generalizations (e.g. [2–11]) of the Gronwall inequality have been extensively discussed. With further study of fractional differential equations, integral inequalities with weakly singular kernels have attracted more and more attention (see [12–20]). In [14], a new method was presented to analyze the nonlinear singular integral inequalities of Henry type:

1.1 $$ u(t)\le a(t)+b(t) \int_{t_{0}}^{t}(t-s)^{\beta-1}s^{\gamma -1}F(s)u(s) \,ds,\quad t\ge0. $$

In 2008, Cheung *et al.* [20] solved the nonlinear weakly singular inequality

1.2 $$\begin{aligned} u^{p}(x,y) \le& a(x,y)+b(x,y) \int_{0}^{x} \int _{0}^{y}\bigl(x^{\alpha}-s^{\alpha} \bigr)^{\beta-1}s^{\gamma-1} \bigl(y^{\alpha}-t^{\alpha} \bigr)^{\beta-1}t^{\gamma-1} \\ &{} \cdot f(s,t)u^{q}(s,t)\,dt\,ds. \end{aligned}$$

On the other hand, since differential equations with maxima of the unknown function [21–26] can be applied in control theory, some significant results for integral inequalities containing the maxima of the unknown function [22, 27–30] have been obtained. The integral inequality with maxima

1.3 $$\begin{aligned}& u(x,y)\leq a(x,y)+ \int_{x_{0}}^{x} \int_{y_{0}}^{y} f(s,t) u^{p}(s,t)\,dt\,ds \\& \hphantom{u(x,y)\leq{}}{} + \int_{\alpha(x_{0})}^{\alpha(x)} \int_{y_{0}}^{y} g(s,t) \Bigl(\max _{\tilde{\eta}\in[s-h,s]}u^{p}(\tilde{\eta },t) \Bigr)\,dt\,ds,\quad x \ge x_{0}, y\ge y_{0}, \\& u(x,y)\leq \psi(x,y), \quad x\in \bigl[\alpha(x_{0})-h, x_{0}\bigr], y\ge y_{0}, \end{aligned}$$

where *f*, *g*, and *ψ* are nonnegative continuous functions and $a(x,y)>0$ is a nondecreasing continuous function, was discussed in [22].

Combining (1.2) with (1.3), we will consider the integral inequality with maxima

1.4 $$ \begin{aligned} &\varphi\bigl(u(x,y)\bigr) \leq a(x,y)+ \sum_{j=1}^{m} \int _{b_{j}(x_{0})}^{b_{j}(x)} \int_{c_{j}(y_{0})}^{c_{j}(y)} \bigl(x^{\alpha_{j}}-s^{\alpha_{j}} \bigr)^{\beta_{j}-1}s^{\gamma_{j}-1}\bigl(y^{\bar {\alpha}_{j}}-t^{\bar{\alpha}_{j}} \bigr)^{\bar{\beta}_{j}-1} t^{\bar{\gamma}_{j}-1} \\ &\hphantom{\varphi(u(x,y)) \leq{}}{}\cdot f_{j}(x,y,s,t) \omega_{j} \bigl(u(s,t)\bigr)\mu_{j} \Bigl(\max_{\tilde{\eta}\in[s-h, s]}g\bigl(u( \tilde{\eta},t)\bigr) \Bigr)\,dt\,ds, \\ &\hphantom{\varphi(u(x,y)) \leq{}}{}(x,y)\in [x_{0},x_{1}) \times[y_{0}, y_{1}), \\ &u(x,y) \leq \psi (x,y), \quad (x,y)\in\bigl[b_{*}(x_{0})-h,x_{0} \bigr]\times [y_{0}, y_{1}), \end{aligned} $$

where *a*, *g*, $\omega_{j}$, $f_{j}$, $b_{j}$, and $c_{j}$ are nonnegative continuous functions, $b_{j}$ and $c_{j}$ are increasing functions and belong to $C^{1}$, $b_{*}(x_{0}):=\min_{1\le j\le{m}}b_{j}(x_{0})$, $h>0$ is a constant. Specially, the monotonicity of *a*, $\omega_{j}$, $\mu_{j}$, $f_{j}$, and *g* is not required. Further, $\omega_{j}$’s are used to construct a sequence of stronger monotonized functions. Then the obtained result is applied for considering the uniqueness of solutions to a boundary value problem of an integral equation with maxima.

## Main result

Let $\mathbb {R}:=(-\infty, +\infty)$, $\mathbb {R}_{+}:=[0,\infty)$, $\Delta:=[x_{0},x_{1})\times[y_{0}, y_{1})$ and $\Xi:= [b_{*}(x_{0})-h,x_{0}]\times[y_{0}, y_{1})$. Define $\Phi_{1}, \Phi_{2}: B\subset\mathbb {R} \rightarrow \mathbb {R}\setminus\{0\}$. As in [4], if $\Phi_{1}/\Phi _{2}$ is nondecreasing on *B*, then $\Phi_{1}\varpropto\Phi_{2}$. Considering inequality (1.4), we make the following assumptions for all $j=1,\ldots,m$:

(A_1_): $b_{j}\in C^{1}([x_{0},x_{1}),\mathbb {R}_{+})$ and $c_{j}\in C^{1}([y_{0},y_{1}), [y_{0},y_{1}))$ are nondecreasing such that $b_{j}(x)\leq x$ and $c_{j}(y)\le y$, and $c_{j}(y_{0})=y_{0}$;
(A_2_): $a\in C(\Delta,\mathbb {R}_{+}) $, $f_{j}\in C(\Delta\times [b_{*}(x_{0}),x_{1})\times[y_{0},y_{1}), \mathbb{R}_{+})$, $\omega_{j},\mu_{j}\in C(\mathbb{R}_{+},\mathbb {R}_{+}) $ with $\omega_{j}(t)>0$, $\mu _{j}(t)>0$ for $t>0$;
(A_3_): $g, \varphi\in C(\mathbb {R}_{+},\mathbb{R}_{+})$ and $\psi\in C(\Xi, \mathbb {R}_{+})$, and *φ* is strictly increasing such that $\lim_{t\rightarrow\infty}\varphi(t)=\infty$;
(A_4_): $\alpha_{j}, \bar{\alpha}_{j}\in(0,1]$, $\beta_{j},\bar{\beta}_{j}\in(0,1)$, $\gamma_{j}>1-\frac{1}{p}$, $\bar{\gamma}_{j}>1-\frac{1}{p}$ such that $\frac{1}{p}+\alpha_{j}(\beta_{j}-1)+\gamma_{j}-1\ge0$, $\frac {1}{p}+\bar{\alpha}_{j}(\bar{\beta}_{j}-1)+\bar{\gamma}_{j}-1\ge0$, $p(\beta_{j}-1)+1>0$, $p(\bar{\beta}_{j}-1)+1>0$, $p>1$.

For those $\omega_{j}$’s, $\mu_{j}$’s given in (A_4_), define $\tilde {\omega}_{j}(t)$ inductively by

2.1 $$ \tilde{\omega}_{j}(t):= \textstyle\begin{cases} \hat{\omega}_{1}(t)\max_{\tau\in[0, t] }\{\hat{\mu }_{1}(\tilde{g}(\tau))\}, & t\ge0, j=1, \\ \max_{\tau\in[0, t] }\{\frac{\hat{\omega}_{j}(\tau)\hat {\mu}_{j+1}(\tilde{g}((\tau))}{\tilde{\omega}_{i-1}(\tau)}\}\tilde{\omega}_{i-1}(t),& t\ge0, j=2,\ldots,m, \end{cases} $$

where $\hat{\omega}_{j}(t):=\max_{\tau\in[0, t] }\{\bar {\omega}_{j}(\tau)\}$, $\hat{\mu}_{j}(t):=\max_{\tau\in[0, t] }\{\bar{\mu}_{j}(\tau)\}$, $\tilde{g}(t):=\max_{\tau\in[0, t] }\{g(\tau)\}$, $\bar{\omega}_{j}(t):=\omega_{j}(t)+\varepsilon_{j}$, $\bar{\mu }_{j}(t):=\mu_{j}(t)+\varepsilon_{j}$ for $t\ge0$, $\epsilon_{j}:= \varepsilon$ if $\omega_{j}(0)=0$ or $:=0$ if $\omega_{j}(0)\neq0$ for all $j=1,2,\ldots,m$, where $\varepsilon>0$ is an arbitrarily given constant.

### Lemma 1

([16])

*Let* *α*, *β*, *γ*, *and* *p* *be positive constants*. *Then*

$$ \int^{t}_{0}\bigl(t^{\alpha}-s^{\alpha} \bigr)^{p(\beta-1)}s^{p(\gamma -1)}\,ds=\frac{t^{\theta}}{\alpha}B\biggl( \frac{p(\gamma-1)+1}{\alpha}, p(\beta-1)+1\biggr),\quad t\in{\mathbb{R}_{+}}, $$

*where* $\theta:=p[\alpha(\beta-1)+\gamma-1]+1$, $B(\xi,\eta)=\int ^{1}_{0}s^{\xi-1}(1-s)^{\eta-1}\,ds$ ($\operatorname{Re} \xi>0$, $\operatorname{Re} \eta>0$) *is the beta function*.

### Lemma 2

*Suppose that*

1. $b_{j}\in C^{1}([x_{0},x_{1}),\mathbb {R}_{+})$ *and* $c_{j}\in C^{1}([y_{0},y_{1}), [y_{0},y_{1}))$ *are nondecreasing with* $b_{j}(x)\leq x$ *on* $[x_{0},x_{1})$, $c_{j}(y)\le y$ *on* $[y_{0},y_{1})$ *and* $c_{j}(y_{0})=y_{0}$ *for all* $j=1,\ldots,m$;
2. $\psi\in C(\Xi,\mathbb {R}_{+})$, $g_{j}\in C(\Delta\times\mathbb {R}^{2}_{+},\mathbb {R}_{+})$ *are nondecreasing functions in* *x* *and* *y* *for all* $j=1,\ldots,m$;
3. $h_{j}, \bar{h}_{j}\in C(\mathbb {R}_{+},\mathbb{R}_{+})$ ($j=1,\ldots,m$) *are all nondecreasing with* $h_{j}(t)>0$, $\bar{h}_{j}(t)>0$ *for* $t>0$, *and* $h_{j}\bar{h}_{j}\propto h_{j+1}\bar{h}_{j+1}$ ($j=1,\ldots,m-1$);
4. $b\in C(\Delta, \mathbb{R}_{+})$, $b_{x}, b_{y}\in(\Delta, \mathbb{R})$, *and* $\max_{s\in[b_{*}(x_{0})-h,x_{0}]}\psi(s,t)\le b(x_{0},t)$ *for all* $t\in [y_{0},y_{1})$.

*If* $u\in C([b_{*}(x_{0})-h,x_{1})\times[y_{0},y_{1}),\mathbb {R}_{+})$ *satisfies the integral inequality*

2.2 $$\begin{aligned}& u(x,y)\leq b(x,y)+\sum_{j=1}^{m} \int _{b_{j}(x_{0})}^{b_{j}(x)} \int_{c_{j}(y_{0})}^{c_{j}(y)} g_{j}(x,y,s,t) \\& \hphantom{u(x,y)\leq{}}{}\times h_{j}\bigl(u(s,t)\bigr)\tilde{{h}}_{j} \Bigl(\max_{\tilde{\eta }\in[s-h, s]}u(\tilde{\eta},t) \Bigr)\,dt\,ds, \quad (x,y) \in\Delta, \\& u(x,y)\leq \psi (x,y), \quad (x,y)\in\Xi, \end{aligned}$$

*then*

2.3 $$ u(x,y)\leq H_{m}^{-1} \biggl(H_{m} \bigl(\eta_{m}(x,y)\bigr)+ \int _{b_{m}(x_{0})}^{b_{m}(x)} \int_{c_{m}(y_{0})}^{c_{m}(y)} g_{m}(x,y,s,t)\,dt\,ds \biggr) $$

*for all* $(x,y)\in[x_{0}, X_{1}^{*}]\times[y_{0},Y_{1}^{*}]$, *where* $H_{j}^{-1}$ *is the inverse of the function*

2.4 $$ H_{j}(t):= \int_{t_{j}}^{t}\frac{ds}{h_{j}(s)\bar{h}_{j}(s)}, \quad t\ge t_{j}>0, j=1,\ldots,m, $$

$t_{j}$ *is a given constant*, *and* $\eta_{j}$ *is defined by*

2.5 $$ \begin{aligned} &\eta_{1}(x,y):=b(x_{0},y_{0})+ \int_{x_{0}}^{x} \bigl\vert b_{x}(s,y_{0}) \bigr\vert \,ds+ \int_{y_{0}}^{y} \bigl\vert b_{x}(x,t) \bigr\vert \, dt, \\ &\eta_{j+1}(x,y):=H_{j}^{-1} \biggl(H_{j}\bigl(\eta_{j}(x,y)\bigr)+ \int_{b_{j}(x_{0})}^{b_{j}(x)} \int_{c_{j}(y_{0})}^{c_{j}(y)}g_{j}(x,y,s,t)dt \,ds \biggr) \end{aligned} $$

*for* $j=1,\ldots,m-1$, *and* $x_{0}\le X_{1}^{*}< x_{1}$, $y_{0}\le Y_{1}^{*}< y_{1}$ *are chosen such that*

2.6 $$ H_{j}\bigl(\eta_{j}\bigl(X^{*}_{1},Y^{*}_{1} \bigr)\bigr)+ \int_{a_{j}(x_{0})}^{a_{j}(X^{*}_{1})} \int_{b_{j}(y_{0})}^{b_{j}(Y^{*}_{1})}g_{j}\bigl(X^{*}_{1},Y^{*}_{1},s,t \bigr)\, dt \,ds\le \int _{u_{j}}^{\infty}\frac{ds}{{h_{j}(s)}\tilde{h}(s)} $$

*for* $j=1,\ldots,m$.

### Proof

Let *b* be positive on Δ. It means that $\eta_{1}(x,y)$ is positive on Δ. Under such a circumstance, $\eta_{1}$ is nondecreasing on Δ and $\eta_{1}(x,y)>0$,

2.7 $$ \eta_{1}(x,y)\ge b(x_{0},y_{0})+ \int_{x_{0}}^{x} b_{x}(s,y_{0}) \,ds+ \int _{y_{0}}^{y}b_{y}(x,t)\, dt=b(x,y). $$

From (2.2) and (2.7), we have

2.8 $$ \begin{aligned} &u(x,y)\leq \eta_{1}(x,y)+ \sum_{j=1}^{m} \int _{b_{j}(x_{0})}^{b_{j}(x)} \int_{c_{j}(y_{0})}^{c_{j}(y)} g_{j}(x,y,s,t) \\ &\hphantom{u(x,y)\leq{}}{}\cdot h_{j}\bigl(u(s,t)\bigr)\bar{h}_{j} \Bigl(\max_{\tilde{\eta}\in[s-h, s]}u(\tilde{\eta},t) \Bigr)\,dt\,ds,\quad (x,y) \in\Delta, \\ &u(x,y)\leq \psi (x,y), \quad (x,y)\in\Xi. \end{aligned} $$

Concerning (2.8), we consider the auxiliary inequality

2.9 $$ \begin{aligned} &u(x,y) \leq \eta_{1}(x,y)+ \sum_{j=1}^{m} \int _{b_{j}(x_{0})}^{b_{j}(x)} \int_{c_{j}(y_{0})}^{c_{j}(y)} g_{j}(\xi,\eta,s,t) \\ &\hphantom{u(x,y) \leq{}}{} \times h_{j}\bigl(u(s,t)\bigr) \bar{h}_{j} \Bigl(\max_{\tilde{\eta}\in[s-h, s]}u(\tilde{\eta},t) \Bigr)\,dt\,ds, \quad (x,y)\in[x_{0},\xi]\times[y_{0}, \eta], \\ &u(x,y) \leq \psi(x,y), \quad (x,y)\in \bigl[b_{*}(x_{0})-h, x_{0}\bigr]\times[y_{0},\eta], \end{aligned} $$

where $x_{0}\leq\xi\le X^{*}_{1}$ and $y_{0}\leq\eta\le Y^{*}_{1}$ are chosen arbitrarily. Having (2.9) we claim

2.10 $$ u(x,y)\leq H_{m}^{-1} \biggl(H_{m} \bigl(\eta_{m}(\xi,\eta,x,y)\bigr)+ \int _{b_{m}(x_{0})}^{b_{m}(x)} \int_{c_{m}(y_{0})}^{c_{m}(y)} g_{m}(\xi,\eta,s,t)\,dt \,ds \biggr) $$

for $x_{0}\le x \le\min\{\xi, X^{*}_{2}\}$, $y_{0}\le y \le\min\{\eta, Y^{*}_{2}\}$, where $\tilde{\eta}_{j}(\xi,\eta,x,y)$ is defined inductively by $\tilde {\eta}_{1}(\xi,\eta,x,y):=\eta_{1}(x,y)$ and

$$ \tilde{\eta}_{j}(\xi,\eta,x,y):= H_{j-1}^{-1} \biggl(H_{j-1}\bigl(\tilde{\eta }_{j-1}(\xi,\eta,x,y)\bigr)+ \int_{b_{j-1}(x_{0})}^{b_{j-1}(x)} \int _{c_{j-1}(y_{0})}^{c_{j-1}(y)} g_{j-1}(\xi,\eta,s,t)\,dt \,ds\biggr) $$

for $j=2,\ldots, m$, and $X^{*}_{2}\in[x_{0},x_{1})$, $Y^{*}_{2}\in[y_{0},y_{1})$ are chosen such that

2.11 $$\begin{aligned} &H_{j}\bigl(\tilde{\eta}_{j}\bigl(\xi, \eta,X^{*}_{2},Y^{*}_{2}\bigr)\bigr)+ \int _{b_{j}(x_{0})}^{b_{j}(X^{*}_{2})} \int_{c_{j}(y_{0})}^{c_{j}(Y^{*}_{2})} g_{j}(\xi,\eta,s,t) \\ &\quad \le \int_{t_{j}}^{\infty}\frac{ds}{{h_{j}(s)}\bar{h}_{j}(s)} \end{aligned}$$

for $j=1,2,\ldots,m$. Note that $X^{*}_{2}\ge X^{*}_{1}$ and $Y^{*}_{2}\ge Y^{*}_{1}$. In fact, both $\tilde{\eta}_{j}(\xi,\eta,x,y)$ and $g_{j}(\xi,\eta,x,y)$ are nondecreasing in *ξ* and *η*. Thus $X^{*}_{2}$, $Y^{*}_{2}$ satisfying (2.11) will get smaller as *ξ*, *η* are chosen larger.

Since $\max_{s\in[b^{*}(x_{0})-h,x_{0}]}\psi(s,t)\le b(x_{0},t)$ and $b(x_{0},t)\le \eta_{1}(x_{0},t)\le\eta_{1}(x,t)$, we obtain

2.12 $$ \max_{s\in[b_{*}(x_{0})-h,x_{0}]}\psi(s,t)\leq\eta _{1}(x,t), \quad (x,t)\in[x_{0},x_{1}) \times[y_{0},y_{1}). $$

First, (2.10) holds for $m=1$. In fact,(2.9) for $m=1$ is written as

2.13 $$ u(x,y)\leq z_{1}(x,y),\quad (x,y)\in \bigl[b_{*}(x_{0})-h, \xi\bigr]\times[y_{0}, \eta], $$

where

2.14 $$ z_{1}(x,y)= \textstyle\begin{cases} \eta_{1}(x,y)+ \int_{b_{1}(x_{0})}^{b_{1}(x)}\int_{c_{1}(y_{0})}^{c_{1}(y)} g_{1}(\xi,\eta,s,t) h_{1}(u(s,t)) \\ \quad {}\times\bar{h}_{1} (\max_{\tilde{\eta}\in[s-h, s]}u(\tilde{\eta},t) )\,dt\,ds, \quad (x,y)\in[x_{0},\xi]\times[y_{0},\eta] \\ \eta_{1}(x_{0},y), \quad (x,y)\in[b_{*}(x_{0})-h, x_{0}]\times[y_{0},\eta], \end{cases} $$

$z_{1}(x,y)$ is a nondecreasing function on $[x_{0}, \xi]\times[y_{0},\eta]$. Then

$$\begin{aligned} \frac{\partial}{\partial x}z_{1}(x,y) =&\frac{\partial }{\partial x} \eta_{1}(x,y)+ \int_{c_{1}(y_{0})}^{c_{1}(y)} g_{1}\bigl(\xi, \eta,b_{1}(x),t\bigr) h_{1}\bigl(u\bigl(b_{1}(x),t \bigr)\bigr) \\ &{}\times\bar{h}_{1} \Bigl(\max_{\tilde{\eta }\in[b_{1}(x)-h, b_{1}(x)]}u(\tilde{ \eta},t) \Bigr)\, dtb'(x) \end{aligned}$$

for all $(x,y)\in[x_{0},\xi]\times[y_{0},\eta] $. We have $0< h_{1}(u(s,t))\bar{h}_{1}(u(s,t))\le h_{1}(z_{1}(s,t))\bar{h}_{1}(z_{1}(s,t)) \le h_{1}(z_{1}(x,y))\bar{h}_{1}(z_{1}(x,y)) $ by (C3) and (2.13) $s\le b_{1}(x)\le x$, $t\le c_{1}(y)\le y$ and both $z_{1}$ and $h_{1}\tilde{h}_{1}$ are nondecreasing. Thus

2.15 $$\begin{aligned}& \frac{\frac{\partial}{\partial x}z_{1}(x,y)}{h_{1}(z_{1}(x,y))\bar{h}_{1}(z_{1}(x,y))} \\& \quad \le \frac{\frac{\partial}{\partial x}\eta _{1}(x,y)}{h_{1}(\eta_{1}(x,y))\bar{h}_{1}(\eta_{1}(x,y))}+\frac {b'(x)}{h_{1}(z_{1}(x,y))\bar{h}_{1}(z_{1}(x,y))} \\& \qquad {}\times \int_{c_{1}(y_{0})}^{c_{1}(y)} g_{1}\bigl(\xi, \eta,b_{1}(x),t\bigr) h_{1}\bigl(u\bigl(b_{1}(x),t \bigr)\bigr)\bar{h}_{1} \Bigl(\max_{\tilde{\eta}\in [b_{1}(x)-h, b_{1}(x)]}u(\tilde{ \eta},t) \Bigr)\,dt \\& \quad \le \frac{\frac{\partial}{\partial x}\eta_{1}(x,y)}{h_{1}(\eta_{1}(x,y))\bar {h}_{1}(\eta_{1}(x,y))}+b'(x) \int_{c_{1}(y_{0})}^{c_{1}(y)} g_{1}\bigl(\xi, \eta,b_{1}(x),t\bigr)\,dt. \end{aligned}$$

Integrating inequality (2.15) from $x_{0}$ to *x*, from (2.4) we get

2.16 $$\begin{aligned} H_{1}\bigl(Z_{1}(x,y)\bigr) \le& H_{1}\bigl( \eta_{1}(x,y)\bigr)+ \int _{x_{0}}^{x}b'(s) \int_{c_{1}(y_{0})}^{c_{1}(y)} g_{1}\bigl(\xi, \eta,b_{1}(s),t\bigr)\,dt\,ds \\ =& H_{1}\bigl(\eta_{1}(x,y)\bigr)+ \int_{b_{1}(x_{0})}^{b_{1}(x)} \int _{c_{1}(y_{0})}^{c_{1}(y)} g_{1}(\xi,\eta,s,t)\,dt \,ds \end{aligned}$$

for all $(x,y)\in[x_{0},\xi]\times[y_{0},\eta]$. From (2.14), (2.16), and the monotonicity of $H^{-1}_{1}$, we have

2.17 $$ u(x,y))\le H^{-1}_{1}\biggl( H_{1}\bigl(\eta_{1}(x,y)\bigr)+ \int_{b_{1}(x_{0})}^{b_{1}(x)} \int _{c_{1}(y_{0})}^{c_{1}(y)} g_{1}(\xi,\eta,s,t)\,dt \,ds\biggr) $$

for $x_{0}\le x\le\xi< X^{*}_{2}$, $Y_{0}\le y \le\eta< Y^{*}_{2}$, implying that (2.7) is true for $m=1$.

Assume that (2.10) holds for $m=k$. Consider

2.18 $$\begin{aligned}& u(x,y) \leq \eta_{1}(x,y)+\sum_{j=1}^{k+1} \int_{b_{j}(x_{0})}^{b_{j}(x)} \int_{c_{j}(y_{0})}^{c_{j}(y)} g_{j}(\xi,\eta,s,t) \\& \hphantom{u(x,y) \leq{}}{}\times h_{j}\bigl(u(s,t)\bigr)\bar{h}_{j} \Bigl(\max_{\tilde{\eta}\in[s-h, s]}u(\tilde{\eta},t)\Bigr)\,dt\,ds,\quad (x,y) \in[x_{0},\xi]\times[y_{0},\eta] \\& u(x,y) \leq \psi (x,y), \quad (x,y)\in\bigl[b_{*}(x_{0})-h, x_{0}\bigr]\times[y_{0},\eta] . \end{aligned}$$

Let

2.19 $$ z_{2}(x,y)= \textstyle\begin{cases} \eta_{1}(x,y) +\sum_{j=1}^{k+1} \int_{b_{j}(x_{0})}^{b_{j}(x)}\int _{c_{j}(y_{0})}^{c_{j}(y)} g_{j}(\xi,\eta, s,t)h_{j}(u(s,t)) \\ \quad {}\cdot\bar{h}_{j}(\max_{\tilde{\eta}\in[s-h, s]}u(\tilde {\eta},t))\,dt\,ds,\quad (x,y)\in[x_{0},\xi]\times[y_{0},\eta], \\ \eta_{1}(x_{0},y),\quad (x,y)\in [b_{*}(x_{0})-h, x_{0}]\times[y_{0},\eta]. \end{cases} $$

Then $z_{2}$ is a nondecreasing function on $[x_{0}, x]\times[y_{0},\eta]$. By (2.19) and the definition of $z_{2}$, it follows that

2.20 $$ u(x,y)\leq z_{2}(x,y),\quad (x,y)\in \bigl[b_{*}(x_{0})-h, \xi\bigr]\times [y_{0}, \eta]. $$

Since $h_{j}\bar{h}_{j}$ is nondecreasing and $z_{2}(x,y)>0$, $b'_{j}(x)\ge 0$, and $b_{j}(x)\le x$, we have

$$\begin{aligned}& \frac{\frac{\partial}{\partial x}z_{2}(x,y)}{h_{1}(z_{2}(x,y))\bar {h}_{1}(z_{2}(x,y))} \\& \quad \le\frac{\frac{\partial}{\partial x}\eta _{1}(x,y)}{h_{1}(z_{2}(x,y))\bar{h}_{1}(z_{2}(x,y))}+\sum_{j=1}^{k+1} \frac{b'_{j}(x)}{ h_{1}(z_{2}(x,y))\bar{h}_{1}(z_{2}(x,y))} \\& \qquad {}\cdot \int_{c_{j}(y_{0})}^{c_{j}(y)}g_{j}\bigl(X,Y,b_{j}(x),t \bigr)h_{j}\bigl(u\bigl(b_{j}(x),t\bigr)\bigr) h_{j}\Bigl(\max_{\xi\in[b_{j}(x)-h,b_{j}(x)]}u(\tilde{\eta},t)\Bigr)\,dt \\& \quad \le\frac{\frac{\partial}{\partial x}\eta_{1}(x,y)}{h_{1}(\eta _{1}(x,y))\bar{h}_{1}(\eta_{1}(x,y))}+\sum_{j=1}^{k+1} \frac{b'_{j}(x)}{ h_{j}(z_{2}(x,y))\bar{h}_{j}(z_{2}(x,y))} \\& \qquad {}\cdot \int_{c_{j}(y_{0})}^{c_{j}(y)}g_{j}\bigl(\xi, \eta,b_{j}(x),t\bigr)h_{j}\bigl(z_{2} \bigl(b_{j}(x),t\bigr)\bigr) \bar{h}_{j}\Bigl(\max _{\tilde{\eta}\in [b_{j}(x)-h,b_{j}(x)]}z_{2}(\tilde{\eta},t)\Bigr)\,dt \\& \quad \le\frac{\frac{\partial}{\partial x}\eta_{1}(x,y)}{h_{1}(\eta _{1}(x,y))\bar{h}_{1}(\eta_{1}(x,y))} +b'_{1}(x) \int_{c_{1}(y_{0})}^{c_{1}(y)}g_{1}\bigl(\xi, \eta,b_{1}(x),t\bigr)\,dt+\sum_{j=1}^{k}b'_{j+1}(x) \\& \qquad {} \cdot \int_{c_{j}(y_{0})}^{c_{j}(y)}g_{j+1}\bigl(\xi,\eta ,b_{j+1}(x),t\bigr)\tilde{h}_{j+1}\bigl(z_{2} \bigl(b_{j+1}(x),t\bigr)\bigr) \hat{h}_{j+1}\Bigl(\max _{\tilde{\eta}\in [b_{j}(x)-h,b_{j}(x)]}z_{2}(\tilde{\eta},t)\Bigr)\,dt \end{aligned}$$

for all $(x,y)\in[x_{0},X_{1}^{*}]\times[y_{0},Y_{1}^{*}]$, where $\tilde {h}_{j+1}(u):=h_{j+1}(u)/h_{1}(u)$, $\hat{h}_{j+1}(u):=\bar{h}_{j+1}(u)/\bar{h}_{1}(u)$, $j=1,\ldots,k$. Integrating the above inequality from $x_{0}$ to *x*, we can obtain

2.21 $$\begin{aligned} H_{1}\bigl(z_{2}(x,y)\bigr) \le& H_{1}\bigl( \eta_{1}(x,y)\bigr)+ \int_{b_{1}(x_{0})}^{b_{1}(x)} \int _{c_{1}(y_{0})}^{c_{1}(y)}g_{1}(\xi,\eta,s,t)\,dt \,ds \\ &{} +\sum_{j=1}^{k} \int_{b_{j+1}(x_{0})}^{b_{j+1}(x)} \int _{c_{j+1}(y_{0})}^{c_{j+1}(y)}g_{j+1}(\xi,\eta,s,t) \tilde{h}_{j+1}\bigl(z_{2}(s,t)\bigr) \\ &{} \cdot\hat{h}_{j+1} \Bigl(\max_{\tilde{\eta}\in [s-h,s]}z_{2}( \tilde{\eta},t) \Bigr)\,dt\,ds \end{aligned}$$

for all $(x,y)\in[x_{0},X]\times[y_{0},Y]$. Let

2.22 $$ \begin{aligned} &\eta(x,y):=H_{1}\bigl(z_{2}(x,y)\bigr), \\ &\varrho_{1}(x,y):=H_{1}\bigl(\eta_{1}(x,y) \bigr)+ \int_{b_{1}(x_{0})}^{b_{1}(x)} \int _{c_{1}(y_{0})}^{c_{1}(y)}g_{1}(\xi,\eta,s,t)\,dt \,ds. \end{aligned} $$

Then inequality (2.21) can be rewritten as

2.23 $$\begin{aligned}& \eta(x,y) \le \varrho_{1}(x,y)+\sum_{j=1}^{k} \int _{b_{j+1}(x_{0})}^{b_{j+1}(x)} \int _{c_{j+1}(y_{0})}^{c_{j+1}(y)}g_{j+1}(\xi,\eta,s,t)\tilde {h}_{j+1}\bigl(H_{1}^{-1}\bigl(z_{2}(s,t) \bigr)\bigr) \\& \hphantom{\eta(x,y) \le{}}{} \cdot\hat{h}_{j+1}\Bigl(\max_{\tilde{\eta}\in [s-h,s]}H_{1}^{-1} \bigl(z_{2}(\tilde{\eta},t)\bigr)\Bigr)\,dt\,ds, \quad (x,y)\in [x_{0},X]\times[y_{0},Y], \\& \eta(x,y) = H_{1}\bigl(\eta_{(}x_{0},y)\bigr)\le \varrho_{1}(x_{0}, y), \quad (x,y)\in\bigl[b_{*}(x_{0})-h, x_{0}\bigr]\times[y_{0},Y], \end{aligned}$$

the same form as (2.9) for $m=k$. By (C3), each $(\bar {h}_{j+1}\circ H_{1}^{-1})(\tilde{h}_{j+1}\circ H_{1}^{-1})$ ($j=1,\ldots,k$) is a nonnegative continuous and increasing function on $\mathbb{R}_{+}$ and positive on $(0,+\infty)$. Moreover, $(\tilde{h}_{j}\circ H_{1}^{-1})\propto(\hat{h}_{j+1}\circ H_{1}^{-1})$ for all $j=2,\ldots, k$. By the inductive assumption, we have

2.24 $$ \eta(x,y)\le \bar{H}_{k+1}^{-1}\biggl( \bar{H}_{k+1}\bigl(\varrho _{k}(x,y)\bigr)+ \int_{b_{k+1}(x_{0})}^{b_{k+1}(x)} \int_{c_{k+1}(y_{0})}^{c_{k+1}(y)} g_{k+1}(\xi,\eta,s,t)\,dt \,ds\biggr) $$

for $x_{0}\le x\le\min\{\xi, X_{3}^{*}\}$, $y_{0}\le y\le\min\{\eta, Y_{3}^{*}\}$, where

2.25 $$ \bar{H}_{j+1}(t):= \int_{\tilde{t}_{j+1}}^{t}\frac{ds}{\tilde {h}_{j+1}(H_{1}^{-1}(s))\hat{h}_{j+1}(H_{1}^{-1}(s))},\quad t>0, $$

$\tilde{t}_{j+1}=H_{1}(t_{j+1})$, $\bar{H}^{-1}_{j+1}$ is the inverse of $\bar{H}_{j+1}$, $j=1,\ldots, k$,

2.26 $$ \varrho_{j+1}(x,y):=\bar{H}^{-1}_{j+1} \biggl(\bar{H}_{j+1}\bigl(\varrho _{j}(x,y)\bigr)+ \int_{b_{j+1}(x_{0})}^{b_{j+1}(x)} \int_{c_{j+1}(y_{0})}^{c_{j+1}(y)}g_{j+1}(\xi,\eta,s,t)\,dt \,ds\biggr), $$

$j=1,\ldots,k-1$, and $X^{*}_{3}$, $Y^{*}_{3}$ are chosen such that

2.27 $$\begin{aligned}& \bar{H}_{j+1}\bigl(\varrho_{j}\bigl(X^{*}_{3},Y^{*}_{3} \bigr)\bigr)+ \int _{b_{j+1}(x_{0})}^{b_{j+1}(X^{*}_{3})} \int_{c_{j+1}(y_{0})}^{c_{j+1}(Y^{*}_{3})}g_{j+1}(\xi,\eta,t,s)\,dt \,ds \\& \quad \le \int_{\tilde{t}_{j+1}}^{H_{1}(\infty)}\frac{ds}{\tilde {h}_{j+1}(H^{-1}_{1}(s))\hat{h}_{j+1}(H_{1}^{-1}(s))},\quad j=1, \ldots,k. \end{aligned}$$

Note that

2.28 $$\begin{aligned} \bar{H}_{j}(t) =& \int_{\tilde{t}_{j}}^{t}\frac {ds}{\tilde{h}_{j}(H_{1}^{-1}(s))\hat{h_{j}}(H_{1}^{-1}(s))} \\ =& \int_{H_{1}(t_{j})}^{t}\frac{h_{1}(H^{-1}_{1}(s))\bar {h}_{1}(H^{-1}_{1}(s))\,ds}{h_{j}(H_{1}^{-1}(s))\bar{h}_{j}(H_{1}^{-1}(s))} \\ =& \int_{H_{1}(t_{j})}^{t}\frac{h_{1}(H^{-1}_{1}(s))\bar {h}_{1}(H^{-1}_{1}(s))\,ds}{h_{j}(H_{1}^{-1}(s))\bar{h}_{j}(H_{1}^{-1}(s))} \\ =& \int_{t_{j}}^{H^{-1}_{1}(t)}\frac{ds}{h_{j}(s)\bar{h}_{j}(s)}=H_{j}\bigl(H^{-1}_{1}(t)\bigr), \quad j=2, \ldots,k+1. \end{aligned}$$

Then, from (2.20), (2.24), and (2.28), we get

2.29 $$\begin{aligned} u(x,y) \le& H^{-1}_{1}\bigl(\eta(x,y)\bigr) \\ \le& H_{k+1}^{-1}\biggl(H_{k+1} \bigl(H^{-1}_{1}\bigl(\varrho_{k}(x,y)\bigr) \bigr) + \int_{b_{k+1}(x_{0})}^{b_{k+1}(x)} \int_{c_{k+1}(y_{0})}^{c_{k+1}(y)} g_{k+1}(\xi,\eta,s,t)\,dt \,ds\biggr) \end{aligned}$$

for $x_{0}\le x\le\min\{X, X_{3}^{*}\}$, $y_{0}\le y\le\min\{Y, Y_{3}^{*}\} $. Let $\tilde{\varrho}_{j}(x,y)=H^{-1}_{1}(\varrho_{j}(x,y))$. Then

2.30 $$\begin{aligned} \tilde{\varrho}_{1}(x,y) =&H_{1}\bigl( \varrho_{1}(x,y)\bigr) \\ =&H^{-1}_{1}\biggl(H_{1}\bigl( \eta_{1}(x,y)\bigr)+ \int_{b_{1}(x_{0})}^{b_{1}(x)} \int _{c_{1}(y_{0})}^{c_{1}(y)}g_{1}(\xi,\eta,s,t)\,dt \,ds\biggr) \\ =&H^{-1}_{1}\biggl(H_{1}\bigl(\tilde{ \eta}_{1}(\xi,\eta,x,y)\bigr)+ \int _{b_{1}(x_{0})}^{b_{1}(x)} \int_{c_{1}(y_{0})}^{c_{1}(y)}g_{1}(\xi,\eta,s,t)\,dt \,ds\biggr) \\ =&\tilde{\eta}_{2}(X,Y,x,y). \end{aligned}$$

Moreover, with the assumption that $\tilde{\varrho}_{k}(x,y)=\tilde {\eta}_{k+1}(\xi,\eta,x,y)$, we get

2.31 $$\begin{aligned} \tilde{\varrho}_{k+1}(x,y) =&H^{-1}_{1}\biggl( \bar {H}^{-1}_{k+1}\biggl(\bar{H}_{k+1}\bigl( \varrho_{k}(x,y)\bigr)+ \int_{b_{k+1}(x_{0})}^{b_{k+1}(x)} \int_{c_{k+1}(y_{0})}^{c_{k+1}(y)}g_{k+1}(\xi,\eta,t,s)\,dt\,ds \biggr)\biggr) \\ =&H^{-1}_{k+1}\biggl(H_{k+1}\bigl(H^{-1}_{1} \bigl(\varrho_{k}(x,y)\bigr)\bigr)+ \int _{b_{k+1}(x_{0})}^{b_{k+1}(x)} \int_{c_{k+1}(y_{0})}^{c_{k+1}(y)}g_{k+1}(\xi,\eta,t,s)\,dt\,ds \biggr) \\ =&H^{-1}_{k+1}\biggl(H_{k+1}\bigl(\tilde{ \varrho}_{k}(x,y)\bigr)+ \int _{b_{k+1}(x_{0})}^{b_{k+1}(x)} \int_{c_{k+1}(y_{0})}^{c_{k+1}(y)}g_{k+1}(\xi,\eta,t,s)\,dt\,ds \biggr) \\ =&H^{-1}_{k+1}\biggl(H_{k+1}\bigl(\tilde{ \eta}_{k+1}(\xi,\eta ,x,y)\bigr)+ \int_{b_{k+1}(x_{0})}^{b_{k+1}(x)} \int_{c_{k+1}(y_{0})}^{c_{k+1}(y)}g_{k+1}(\xi,\eta,t,s)\,dt\,ds \biggr) \\ =&\tilde{\eta}_{k+2}(\xi,\eta,x,y). \end{aligned}$$

This proves that

2.32 $$ \tilde{\varrho}_{j}(x,y)=\tilde{\eta}_{j+1}( \xi,\eta, x,y),\quad j=1,\ldots, k . $$

Therefore, (2.27) becomes

2.33 $$\begin{aligned}& H_{j+1}\bigl(\tilde{\eta}_{j+1}\bigl(\xi, \eta,X^{*}_{3},Y^{*}_{3}\bigr) \bigr)+ \int _{b_{j+1}(x_{0})}^{b_{j+1}(X^{*}_{3})} \int_{c_{j+1}(y_{0})}^{c_{j+1}(Y^{*}_{3})}g_{j+1}(\xi,\eta,t,s)\,dt \,ds \\& \quad \le \int_{\tilde{t}_{j+1}}^{H_{1}(\infty)}\frac{ds}{\tilde {h}_{j+1}(H^{-1}_{1}(s))\hat{h}_{j+1}(H_{1}^{-1}(s))} \\& \quad = \int_{t_{j+1}}^{\infty}\frac{ds}{h_{j+1}(s)\bar{h}_{j+1}(s)},\quad j=1, \ldots,k, \end{aligned}$$

which implies that $X^{*}_{2}=X^{*}_{3}$, $\xi\le X^{*}_{3}$, $Y^{*}_{2}=Y^{*}_{3}$, $\eta\le Y^{*}_{3}$. From (2.29) we obtain

$$ u(x,y)\le H_{k+1}^{-1}\biggl(H_{k+1}\bigl(\tilde{ \eta}_{k+1}(\xi,\eta,x,y)\bigr)+ \int _{b_{k+1}(x_{0})}^{b_{k+1}(x)} \int_{c_{k+1}(y_{0})}^{c_{k+1}(y)} g_{k+1}(\xi,\eta,s,t)\,dt \,ds\biggr) $$

for $x_{0}\le x\le \min\{X,X_{2}^{*}\}$, $y_{0}\le y\le \min\{Y,Y_{2}^{*}\}$. This proves (2.10) by induction.

Taking $x=\xi,\eta$, $y=\xi,\eta$ in (2.10), we have

2.34 $$\begin{aligned} u(\xi,\eta) \leq&H_{m}^{-1} \biggl(H_{m}\bigl( \tilde{\eta}_{m}(\xi,\eta ,\xi,\eta)\bigr)+ \int_{b_{m}(x_{0})}^{b_{m}(X)} \int _{c_{m}(y_{0})}^{c_{m}(\eta)} g_{m}(\xi,\eta,s,t)\,dt \,ds \biggr) \\ = &H_{m}^{-1} \biggl(H_{m}\bigl( \eta_{m}(\xi,\eta)\bigr)+ \int _{b_{m}(x_{0})}^{b_{m}(\xi)} \int_{c_{m}(y_{0})}^{c_{m}(\eta)} g_{m}(\xi,\eta,s,t)\,dt \,ds \biggr) \end{aligned}$$

for $x_{0}\le\xi\le X^{*}_{1}$, $y_{0}\le\eta\le Y^{*}_{1}$, since $x^{*}_{2}\ge X^{*}_{1}$, $Y^{*}_{2}\ge Y^{*}_{1}$ and $\tilde{\eta}_{m}(\xi,\eta,\xi,\eta)= \eta_{m}(\xi,\eta)$. Since *ξ*, *η* are arbitrary, replacing *ξ* and *η* with *x* and *y*, respectively, we have

2.35 $$ u(x,y) \le H_{m}^{-1} \biggl(H_{m}\bigl( \eta_{m}(x,y)\bigr)+ \int _{b_{m}(x_{0})}^{b_{m}(x)} \int_{c_{m}(y_{0})}^{c_{m}(y)} g_{m}(x,y,s,t)\,dt\,ds \biggr) $$

for all $(x,y)\in[x_{0}, X^{*}_{1}]\times[y_{0},Y^{*}_{1}]$.

Let $b(x,y)=0$ for some $(x,y)\in\Delta$. Let $\eta_{1,\epsilon }(x,y):=r_{1}(x,y)+\epsilon$ for any $\epsilon>0$. Then $\eta_{1,\epsilon}(x,y)>0$. Using the same arguments as above, where $\eta_{1}(x,y)$ is replaced with $\eta_{1,\epsilon}(x,y)$, we get

$$ u(x,y)\leq H_{m}^{-1}\biggl(H_{m}\bigl( \eta_{n,\epsilon}(x,y)\bigr)+ \int _{b_{m}(x_{0})}^{b_{m}(x)} \int_{c_{m}(y_{0})}^{c_{m}(y)} g_{m}(x,y,s,t)\,dt\,ds \biggr) $$

for $x_{0}\le x\le X^{*}_{1}$, $y_{0}\le Y^{*}_{1}$. Then consider the continuity of $\eta_{i,\epsilon}$ in *ϵ* and the continuity of $H_{j}$ and $H_{j}^{-1}$ for $j=1,\ldots, m$, and let $\epsilon\rightarrow0^{+}$. Then we obtain (2.7). This completes the proof. □

### Theorem 2.1

*Suppose that* (A_1_)*–*(A_4_) *hold*. $\max_{s\in [b_{*}(x_{0})-h,x_{0}]}\psi(s,y)\leq\varphi^{-1}( (1+m)^{1-1/q}a(x_{0}, y))$ *for* $y\in[y_{0},y_{1})$ *and* $u\in C([b_{*}(x_{0})-h,x_{1})\times[y_{0},y_{1}),\mathbb {R}_{+})$ *are satisfied* (1.4). *Then*, *for all* $(x,y)\in[x_{0}, X_{1})\times[y_{0},Y_{1})$, *we have*

2.36 $$ u(x,y)\leq\varphi^{-1}\biggl(\biggl(W_{m}^{-1} \bigl(W_{m}\bigl(r_{m}(x,y)\bigr)\bigr)+ \int_{\alpha _{m}(x_{0})}^{\alpha_{m}(x)} \int_{\beta_{m}(y_{0})}^{\beta_{m}(y)} \tilde{f}_{m}(x,y,s,t)\,dt \,ds\biggr)^{1/q}\biggr), $$

*where* $W_{j}^{-1}$*is the inverse of the function*

2.37 $$ W_{j}(t):= \int_{t_{j}}^{t}\frac{ds}{\tilde{\omega}^{q}_{j}(\varphi ^{-1}(s^{1/q}))}, \quad t\ge t_{j}>0, j=1,\ldots,m. $$

*In* (2.36) *and* (2.37), $t_{j}$ *is a given constant*, $\frac {1}{p}+\frac{1}{q}=1$, $\tilde{\omega}_{j}$ ($j=1,2,\ldots,m$) *are defined by* (2.1),

2.38 $$\begin{aligned}& r_{1}(x,y) := (1+m)^{q-1}\Bigl(\max_{(\tau,\xi)\in [x_{0}, x]\times[y_{0},y] } \bigl\{ a(\tau,\xi)\bigr\} \Bigr)^{q}, \\& r_{j}(x,y): = W_{j-1}^{-1} \biggl[W_{j-1} \bigl(r_{j-1}(x,y)\bigr)+ \int_{b_{i-1}(x_{0})}^{b_{i-1}(x)} \int _{c_{i-1}(y_{0})}^{c_{i-1}(y)} \tilde{f}_{i-1}(x,y,s,t) \,dt\,ds \biggr], \\& \quad j=2,\ldots, m, \end{aligned}$$

2.39 $$\begin{aligned}& \begin{aligned}[b] &\tilde{f}_{j}(x,y,s,t):=(1+m)^{q-1} \bigl({M_{j}} x^{\theta_{j}}{\bar{M}_{j}} y^{\bar{\theta}_{j}}\bigr)^{q/p}\Bigl(\max_{(\iota,\xi)\in[x_{0}, x ]\times[y_{0},y]}f_{j}( \iota,\xi,s,t)\Bigr)^{q}, \\ &\quad (x,y)\in [x_{0},x_{1})\times[y_{0},y_{1}), \end{aligned} \end{aligned}$$

2.40 $$\begin{aligned}& \begin{aligned} &M_{j}=\alpha_{j}^{-1}B \biggl(\frac{p(\gamma_{j}-1)+1}{\alpha_{j}}, p(\beta_{j}-1)+1\biggr), \\ &\bar{M}_{j}=\bar{\alpha}_{j}^{-1}B\biggl( \frac{p(\bar{\gamma }_{j}-1)+1}{\bar{\alpha}_{j}}, p(\beta_{j}-1)+1\biggr), \\ &\theta_{j}=p\bigl(\alpha_{j}(\beta_{j}-1)+ \gamma_{j}-1\bigr)+1, \\ &\bar{\theta}_{j}=p\bigl(\bar{\alpha}_{j}(\bar{ \beta}_{j}-1)+\bar{\gamma }_{j}-1\bigr)+1, \quad j=1, \ldots,m, \end{aligned} \end{aligned}$$

$X_{1}\in[x_{0}, x_{1})$, $Y_{1}\in[y_{0}, y_{1})$ *are chosen such that*

2.41 $$ W_{j}\bigl(r_{j}(X_{1},Y_{1}) \bigr)+ \int_{b_{j}(x_{0})}^{b_{j}(X_{1})} \int _{c_{j}(y_{0})}^{c_{j}(Y_{1})} \tilde{f}_{j}(x,y,s,t) \,dt\,ds\le \int_{t_{j}}^{\infty}\frac{ds}{\tilde {\omega}^{q}_{j}(\varphi^{-1}(s^{1/q}))} $$

*for* $j=1,\ldots,m$.

### Proof

Above all, we monotonize functions $f_{j}$, $\omega_{j}$, $\mu_{j}$, *g*, and *a* in (1.4). Let

$$ \hat{a}(x,y): = \max_{(\tau,\xi)\in[x_{0}, x]\times[y_{0},y] }\bigl\{ a(\tau,\xi) \bigr\} ,\quad (x,y)\in[x_{0},x_{1})\times[y_{0},y_{1}), $$

which is increasing in *x* and *y*. The sequence $\{\tilde{\omega}_{j}\}$, defined by $\omega_{j}(s)$ and $\mu_{j}(s)$ in (2.1), consists of nonnegative and nondecreasing functions on $\mathbb{R}_{+} $ and satisfies

2.42 $$ \omega_{j}(t)\le\hat{ {\omega}}_{j}(t),\qquad \mu_{j}(t)\le\hat{\mu}_{j}(t),\qquad \hat{\omega}_{j}(t) \hat{{\mu}}_{j}\bigl(\tilde{g}(t)\bigr)\le\tilde{\omega }_{j}(t), \quad j=1,\ldots,m. $$

Moreover, because the ratios ${\tilde{\omega}_{j+1}}/{\tilde{\omega }_{j}}$ ($j=1,\ldots,m-1$) are all nondecreasing, we have

2.43 $$ \tilde{\omega}_{j}\varpropto\tilde{\omega}_{j+1}, \quad j=1,2,\ldots,m-1. $$

Let

2.44 $$ \hat{f}_{j}(x,y,s,t) :=\max_{(\iota,\xi)\in[x_{0}, x ]\times[y_{0},y]}f_{j}( \iota ,\xi,s,t), $$

which are increasing in *x* and *y* and satisfy $\tilde {f}_{j}(x,y,s,t)\geq f_{j}(x,y,s,t)\geq0$ for $j=1,2,\ldots,m$. Since *g̃* is nondecreasing, we obtain

2.45 $$ \max_{\tilde{\eta}\in[s-h,s]} g\bigl(u(\xi,y)\bigr)\le\max _{\tilde{\eta}\in[s-h,s]} \tilde{g}\bigl(u(\xi,y)\bigr) \le\tilde{g}\Bigl(\max _{\tilde{\eta}\in[s-h,s]} u(\xi,y)\Bigr) $$

for all $(s,y)\in[b_{*}(x_{0}), x_{1})\times[y_{0},y_{1})$. From (1.4), (2.42), (2.45), and the definition of $\hat{f}_{j}$, we can obtain

2.46 $$ \begin{aligned} &\varphi\bigl(u(x,y)\bigr)\leq \hat{a}(x,y)+\sum_{j=1}^{m} \int _{b_{j}(x_{0})}^{b_{j}(x)} \int_{c_{j}(y_{0})}^{c_{j}(y)} \bigl(x^{\alpha_{j}}-s^{\alpha_{j}} \bigr)^{\beta_{j}-1}s^{\gamma_{j}-1}\bigl(y^{\bar {\alpha}_{j}}-t^{\bar{\alpha}_{j}} \bigr)^{\bar{\beta}_{j}-1} t^{\bar{\gamma}_{j}-1} \\ &\hphantom{\varphi(u(x,y))\leq{}}{}\times\hat{f}_{j}(x,y,s,t) \hat{ \omega}_{j}\bigl(u(s,t)\bigr)\hat{\mu}_{j}\Bigl(\tilde{g} \Bigl(\max_{\tilde {\eta}\in[s-h,s]}u(\tilde{\eta},t)\Bigr)\Bigr)\,dt\,ds, \quad \\ &\hphantom{\varphi(u(x,y))\leq{}}{}(x,y)\in[x_{0},x_{1}) \times[y_{0}, y_{1}), \\ &u(x,y) \leq \psi (x,y),\quad (x,y)\in\bigl[b_{*}(x_{0})-h,x_{0} \bigr]\times [y_{0}, y_{1}). \end{aligned} $$

Let $\frac{1}{p}+\frac{1}{q}=1$, $p>1$, then $q>0$. By Lemma 1, Hölder’s inequality, (A_4_) and (2.46), we obtain, for all $(x,y)\in[x_{0},x_{1})\times[y_{0}, y_{1})$,

2.47 $$\begin{aligned}& \varphi\bigl(u(x,y)\bigr) \\& \quad \leq \hat{a}(x,y)+\sum _{j=1}^{m} \biggl( \int _{b_{j}(x_{0})}^{b_{j}(x)} \int_{c_{j}(y_{0})}^{c_{j}(y)} \bigl(x^{\alpha_{j}}-s^{\alpha_{j}} \bigr)^{p(\beta_{j}-1)}s^{p(\gamma_{j}-1)}\bigl(y^{\bar {\alpha}_{j}}-t^{\bar{\alpha}_{j}} \bigr)^{p(\bar{\beta}_{j}-1)} t^{(\bar{\gamma}_{j}-1)}\,dt\,ds\biggr)^{1/p} \\& \qquad {}\cdot\biggl( \int _{b_{j}(x_{0})}^{b_{j}(x)} \int_{c_{j}(y_{0})}^{c_{j}(y)}\hat{f}^{q}_{j}(x,y,s,t) \hat{\omega}^{q}_{j}\bigl(u(s,t)\bigr) \Bigl(\hat{ \mu}_{j}\Bigl(\tilde{g}\Bigl(\max_{\tilde{\eta}\in[s-h,s]}u(\tilde{ \eta},t)\Bigr)\Bigr)\Bigr)^{q} \,dt\,ds\biggr)^{1/q} \\& \quad \leq \hat{a}(x,y)+\sum_{j=1}^{m} \biggl( \int _{0}^{x} \int_{0}^{y} \bigl(x^{\alpha_{j}}-s^{\alpha_{j}} \bigr)^{p(\beta_{j}-1)}s^{p(\gamma_{j}-1)}\bigl(y^{\bar {\alpha}_{j}}-t^{\bar{\beta}_{j}} \bigr)^{p(\bar{\gamma}_{j}-1)} t^{{p(\bar{\gamma}_{j}-1)}}\,dt\,ds\biggr)^{1/p} \\& \qquad {}\cdot\biggl( \int_{b_{j}(x_{0})}^{b_{j}(x)} \int_{c_{j}(y_{0})}^{c_{j}(y)}\hat {f}^{q}_{j}(x,y,s,t) \hat{\omega}^{q}_{j}\bigl(u(s,t)\bigr) \Bigl(\hat{ \mu}_{j}\Bigl(\tilde{g}\Bigl(\max_{\tilde{\eta}\in[s-h,s]}u(\tilde{ \eta},t)\Bigr)\Bigr)\Bigr)^{q}dtds\biggr)^{1/q} \\& \quad \leq \hat{a}(x,y)+ \sum_{j=1}^{m} \bigl(M_{j}x^{\theta_{j}}\bar{M}_{j}y^{\bar{\theta}_{j}} \bigr)^{1/p}\biggl( \int _{b_{j}(x_{0})}^{b_{j}(x)} \int_{c_{j}(y_{0})}^{c_{j}(y)}\hat{f}^{q}_{j}(x,y,s,t) \\& \qquad {} \cdot\hat{\omega}^{q}_{j}\bigl(u(s,t)\bigr) \Bigl( \hat{\mu}_{j}\Bigl(\tilde{g}\Bigl(\max_{\tilde{\eta}\in[s-h,s]}u( \tilde{\eta},t)\Bigr)\Bigr)\Bigr)^{q} \,dt\,ds\biggr)^{1/q}, \end{aligned}$$

where $0\le b_{j}(t)\le t $, $0\le c_{j}(t)\le t$, $M_{j}$, $\bar {M}_{j}$, $\theta_{j}$, and $\bar{\theta}_{j}$ are given by (2.40) for $j=1,\ldots,m$.

By Jensen’s inequality and (2.47), we get, for all $(x,y)\in \Delta$,

2.48 $$\begin{aligned} \varphi^{q}\bigl(u(x,y)\bigr) \leq& (1+m)^{q-1}\Biggl( \hat{a}^{q}(x,y)+ \sum_{j=1}^{m} \bigl(M_{j}x^{\theta_{j}}\bar{M}_{j}y^{\bar{\theta}_{j}} \bigr)^{q/p} \int _{b_{j}(x_{0})}^{b_{j}(x)} \int_{c_{j}(y_{0})}^{c_{j}(y)}\hat{f}^{q}_{j}(x,y,s,t) \\ &{} \times\hat{\omega}^{q}_{j}\bigl(u(s,t)\bigr) \Bigl( \hat{\mu}_{j}\Bigl(\tilde{g}\Bigl(\max_{\tilde{\eta}\in[s-h,s]}u( \tilde{\eta},t)\Bigr)\Bigr)\Bigr)\Biggr)^{q} \,dt\,ds. \end{aligned}$$

Then, from (2.38), $r_{1}$ is increasing on Δ. Then, by the definition of $r_{1}$ and $\tilde{f}_{j}$, from (2.48) we have

2.49 $$\begin{aligned} \varphi^{q}\bigl(u(x,y)\bigr) \leq& r_{1}(x,y)+ \sum _{j=1}^{m} \int _{b_{j}(x_{0})}^{b_{j}(x)} \int_{c_{j}(y_{0})}^{c_{j}(y)}\tilde{f}_{j}(x,y,s,t) \hat{\omega}^{q}_{j}\bigl(u(s,t)\bigr) \\ &{}\cdot\Bigl(\hat{\mu}_{j}\Bigl(\tilde{g}\Bigl(\max _{\tilde{\eta}\in [s-h,s]}u(\tilde{\eta},t)\Bigr)\Bigr)\Bigr)^{q}\,dt \,ds, \quad (x,y)\in\Delta. \end{aligned}$$

According to (2.49), we consider the inequalities

2.50 $$ \begin{aligned} &\varphi^{q}\bigl(u(x,y)\bigr) \leq r_{1}(X,Y)+\sum_{j=1}^{m} \int_{b_{j}(x_{0})}^{b_{j}(x)} \int_{c_{j}(y_{0})}^{c_{j}(y)} \tilde{f}_{j}(X,Y,s,t) \\ &\hphantom{\varphi^{q}(u(x,y)) \leq{}}{}\cdot\hat{\omega}^{q}_{j}\bigl(u(s,t) \bigr) \Bigl(\hat{\mu}_{j}\Bigl(\hat{g}\Bigl(\max_{\tilde{\eta}\in[\tilde{\eta}-h,s]}u( \tilde{\eta },t)\Bigr)\Bigr)\Bigr)^{q} \,dt\,ds, \\ &\hphantom{\varphi^{q}(u(x,y)) \leq{}}{} (x,y) \in[x_{0},X]\times[y_{0},Y], \\ &u(x,y) \leq \psi(x,y), \quad (x,y)\in \bigl[b_{*}(x_{0})-h,x_{0} \bigr]\times[y_{0},Y], \end{aligned} $$

where $x_{0}\leq X\le X_{1}$ and $y_{0}\leq Y\le Y_{1}$ are chosen arbitrarily.

Since $\max_{s\in[b_{*}(x_{0})-h,x_{0}]}\psi(s,y)\leq\varphi ^{-1}((1+m)^{1-1/q}a(x_{0},y)) $ for $y\in[y_{0},y_{1})$, $a(x_{0},y)\le\hat{ a}(x_{0},y)$, we have $\max_{s\in[b_{*}(x_{0})-h,x_{0}]}\psi(s,y)\leq\varphi ^{-1}(r^{1/q}_{1}(X,Y))$, $y\in[y_{0},Y]$. Define a function $z(x,y): [b_{*}(x_{0})-h, X)\times[y_{0},Y)\rightarrow \mathbb {R}_{+}$ by

$$ z(x,y)= \textstyle\begin{cases} r_{1}(X,Y)+\sum_{j=1}^{m} \int_{b_{j}(x_{0})}^{b_{j}(x)}\int _{c_{j}(y_{0})}^{c_{j}(y)}\tilde{f}_{j}(X,Y,s,t) \hat{\omega}^{q}_{j}(u(s,t)) \\ \quad {}\times (\hat{\mu}_{j}(\hat{g}(\max_{\tilde{\eta}\in [s-h,s]}u(\tilde{\eta},t))))^{q}\,dt\,ds, \quad (x,y)\in[x_{0},X]\times [y_{0},Y], \\ r_{1}(X,Y), \quad (x,y)\in[b_{*}(x_{0})-h,x_{0}]\times [y_{0}, Y]. \end{cases} $$

Clearly, $z(x,y)$ is increasing in *x*. By the definition of $z(x,y)$ and (2.50), we have

2.51 $$ u(x,y)\leq\varphi^{-1}\bigl(z^{1/q}(x,y) \bigr), \quad (x,y)\in\bigl[b_{*}(x_{0})-h, X\bigr]\times[y_{0},Y]. $$

Since $\varphi(t)$ is strictly increasing and $z(x,y)$ is nondecreasing, from (2.51) we get, for $(s,y)\in[b_{*}(x_{0}), X]\times[y_{0},Y]$,

2.52 $$ \max_{\xi\in[s-h, s]} u(\xi,y) \leq \max _{\xi\in[s-h, s]} \varphi^{-1}\bigl(z^{1/q}(\xi,y) \bigr) \leq \varphi^{-1}\bigl(z^{1/q}(s,y)\bigr). $$

From the definition of $z(x,y)$, (2.42), (2.51), and (2.52), it follows that

2.53 $$\begin{aligned}& z(x,y) \leq r_{1}(X,Y)+\sum_{j=1}^{m} \int _{b_{j}(x_{0})}^{b_{j}(x)} \int_{b_{j}(y_{0})}^{b_{j}(y)} \tilde{f}_{j}(X,Y,s,t) \hat{\omega}^{q}_{j}\bigl(\varphi^{-1} \bigl(z^{1/q}(s,t)\bigr)\bigr) \\& \hphantom{z(x,y) \leq{}} {}\cdot\Bigl(\hat{\mu}_{j}\Bigl(\hat{g}\Bigl(\max _{\tilde{\eta}\in[s-h, s]} \varphi^{-1}\bigl(z^{1/q}(\tilde{ \eta},t)\bigr)\Bigr)\Bigr)\Bigr)^{q}\,dt\,ds \\& \hphantom{z(x,y) } \leq r_{1}(X,Y)+\sum_{j=1}^{m} \int _{b_{j}(x_{0})}^{b_{j}(x)} \int_{b_{j}(y_{0})}^{b_{j}(y)} \tilde{f}_{j}(X,Y,s,t) \hat{\omega}^{q}_{j}\bigl(\varphi^{-1} \bigl(z^{1/q}(s,t)\bigr)\bigr) \\& \hphantom{z(x,y) \leq{}} {}\cdot\bigl(\hat{\mu}_{j}\bigl(\tilde{g}\bigl( \varphi^{-1}\bigl(z^{1/q}(s,t)\bigr)\bigr)\bigr) \bigr)^{q}\,dt\,ds \\& \hphantom{z(x,y) } \leq r_{1}(X,Y)+\sum_{j=1}^{m} \int _{b_{j}(x_{0})}^{b_{j}(x)} \int_{b_{j}(y_{0})}^{b_{j}(y)} \tilde{f}_{j}(X,Y,s,t) \tilde{\omega}^{q}_{j}\bigl(\varphi^{-1} \bigl(z^{1/q}(s,t)\bigr)\bigr) \\& \hphantom{z(x,y) \leq{}} {} \cdot\vartheta_{j}\Bigl(\max_{\tilde{\eta}\in[s-h, s]} u(\tilde {\eta},t)\Bigr)\,dt\,ds, \quad (x,y)\in[x_{0}, X] \times[y_{0},Y], \\& z(x,y) \leq r_{1}(X,Y), \quad (x,y)\in\bigl[b_{*}(x_{0})-h,x_{0} \bigr]\times[y_{0}, Y], \end{aligned}$$

where $\vartheta_{j}(t)\equiv1$, $t\ge0$.

Let $v(t):=\varphi^{-1}(t^{1/q})$, which is a continuous and increasing function on $\mathbb {R}_{+}$. Thus $\tilde{\omega }^{q}_{j}(h(t))$ ($j=1,\ldots, m$) are continuous and increasing on $\mathbb {R}_{+}$ and satisfy $\tilde{\omega}_{j}(v(t))>0 $ for $t>0$. Moreover, since $\tilde {\omega}_{j}(t)\propto\tilde{\omega}_{j+1}(t)$, $\tilde{\omega}^{q}_{j+1}(v(t))/\tilde{\omega}^{q}_{j}(v(t))$ are continuous and increasing on $\mathbb {R}_{+}$ and positive on $(0,\infty)$, then $(\tilde{\omega}_{j}\circ v)\vartheta_{j}\propto (\tilde{\omega}_{j+1}\circ v)\vartheta_{j+1}$ for $j=1,2,\ldots,m-1$.

Applying Lemma 2 to specified $g_{j}(x,y,s,t)=\tilde{f}_{j}(X,Y,s,t)$, $h_{j}(t)=\tilde{\omega}^{q}_{j}(\varphi^{-1}(t^{1/q}))$, $\bar{h}_{j}(t)=\vartheta_{j}(t)\equiv1$ ($j=1,2,\ldots,m$), and (2.53), we obtain

2.54 $$\begin{aligned} z(x,y) \le& W_{m}^{-1}\biggl[W_{n}\bigl(\tilde {r}_{m}(X,Y,x,y)\bigr) \\ &{} + \int_{b_{m}(x_{0})}^{b_{m}(x)} \int_{c_{m}(y_{0})}^{c_{m}(y)} \tilde{ f}_{m}(X,Y,s,t) \,dt\,ds\biggr] \end{aligned}$$

for $x_{0}\le x \le\min\{X, X_{2}\}$, $y_{0}\le y \le\min\{Y, Y_{2}\}$, where $\tilde{r}_{j}$ is defined inductively by $\tilde{r}_{1}(X,Y, x,y):=\gamma _{1}(X,Y)$ and

$$ \tilde{r}_{j}(X,Y,x,y):= W_{i-1}^{-1} \biggl(W_{i-1}\bigl(\tilde {r}_{i-1}(X,Y,x,y)\bigr)+ \int_{b_{i-1}(x_{0})}^{b_{i-1}(x)} \int _{c_{i-1}(y_{0})}^{c_{i-1}(y)} \tilde{f}_{i-1}(X,Y,s,t) \,dt\,ds\biggr) $$

for $j=2,\ldots, m$, and $\bar{X}_{1}$, $\bar{Y}_{1}$ are chosen such that

2.55 $$\begin{aligned}& W_{j}\bigl(\tilde{r}_{j}(X,Y,\bar{X}_{1}, \bar{Y}_{1})\bigr)+ \int _{b_{j}(x_{0})}^{b_{j}(X_{2})} \int_{c_{j}(y_{0})}^{c_{j}(\bar{Y}_{1})} \tilde{f}_{j}(X,Y,s,t) \\& \quad \le \int_{t_{j}}^{\infty}\frac{ds}{\tilde{\omega}^{q}_{j}(\varphi ^{-1}(s^{1/q}))} \end{aligned}$$

for $j=1,\ldots,m$.

Note that $X_{2}\ge X_{1}$ and $Y_{2}\ge Y_{1}$. In fact, both $\tilde {r}_{j}(X,Y,x,y)$ and $\tilde{f}_{j}(X,Y,x,y)$ are increasing in *X* and *Y*. Thus $X_{2}$, $Y_{2}$ satisfying (2.55) get smaller as *X*, *Y* are chosen larger.

According to (2.51) and (2.54),

2.56 $$\begin{aligned} u(x,y) \le& \varphi^{-1}\biggl(W_{m}^{-1} \biggl(W_{n}\bigl(\tilde {r}_{m}(X,Y,x,y)\bigr) \\ &{}+ \int_{\alpha_{m}(x_{0})}^{\alpha_{m}(x)} \int_{\beta _{m}(y_{0})}^{\beta_{m}(y)} \tilde{ f}_{m}(X,Y,s,t) \,dt\,ds\biggr)\biggr) \end{aligned}$$

for $x_{0}\le x \le\min\{X, X_{2}\}$, $y_{0}\le y \le\min\{Y, Y_{2}\}$.

Taking $x=X$, $y=Y$ in (2.56), we have

2.57 $$\begin{aligned} u(X,Y) \le& \varphi^{-1}\biggl(W_{m}^{-1} \biggl(W_{n}\bigl(\tilde {r}_{m}(X,Y,X,Y)\bigr) \\ &{}+ \int_{b_{m}(x_{0})}^{b_{m}(X)} \int_{c_{m}(y_{0})}^{c_{m}(Y)} \tilde{ f}_{m}(X,Y,s,t) \,dt\,ds\biggr)\biggr) \end{aligned}$$

for $x_{0}\le X\le X_{1}$, $y_{0}\le Y\le Y_{1}$. It is easy to verify $\tilde {r}_{m}(X,Y,X,Y)= r_{m}(X,Y)$. Thus, (2.57) can be written as

2.58 $$\begin{aligned} u(X,Y) \le& \varphi^{-1}\biggl(W_{m}^{-1} \biggl(W_{n}\bigl(r_{m}(X,Y)\bigr) \\ &{}+ \int_{b_{m}(x_{0})}^{b_{m}(X)} \int_{c_{m}(y_{0})}^{c_{m}(Y)} \tilde{ f}_{m}(X,Y,s,t) \,dt\,ds\biggr)\biggr). \end{aligned}$$

Since *X*, *Y* are arbitrary, replacing *Y* and *X* with *y* and *x*, respectively, we have

2.59 $$\begin{aligned} u(x,y) \le& \varphi^{-1}\biggl(W_{m}^{-1} \biggl(W_{n}\bigl(r_{m}(x,y)\bigr) \\ &{}+ \int_{b_{m}(x_{0})}^{b_{m}(x)} \int_{c_{m}(y_{0})}^{c_{m}(y)} \tilde{ f}_{m}(x,y,s,t) \,dt\,ds\biggr)\biggr) \end{aligned}$$

for all $(x,y)\in[x_{0}, X^{*}_{1}]\times[y_{0},Y^{*}_{1}]$.

This completes the proof. □

### Theorem 2.2

*We make the following assumptions*:

(S_1_): $c(x,y)\in C(\Delta, \mathbb{R}_{+}) $ *and* $b_{j}\in C^{1}([x_{0},x_{1}),\mathbb {R}_{+})$, *and* $c_{j}\in C^{1}([y_{0},y_{1}), [y_{0},y_{1}))$ *are nondecreasing with* $b_{j}(x)\leq x$ *on* $[x_{0},x_{1})$ *and* $c_{j}(y)\le y$ *on* $[y_{0},y_{1})$, *and* $c_{j}(y_{0})=y_{0}$ *for* $j=1,\ldots,m$;
(S_2_): $\hat{\psi}\in C(\Xi,\mathbb {R}_{+})$, $\hat{g}_{j}\in C(\Delta\times[b_{*}(x_{0}),x_{1})\times[y_{0},y_{1}),\mathbb {R}_{+})$ ($j=1,2,\ldots, m$);
(S_3_): $\phi_{j}, \hat{\phi}_{j}\in C(\mathbb {R}_{+},\mathbb{R}_{+})$ ($j=1,\ldots ,m$) *are all nondecreasing with* $\{\phi_{j},\hat{\phi}_{j}\}(t)>0$ *for* $t>0$, *and* $\phi_{j}\hat{\phi}_{j}\propto\phi_{j+1}\hat{\phi}_{j+1}$ ($j=1,\ldots,m-1$);
(S_4_): $k\ge1$, $\alpha_{j}, \bar{\alpha}_{j}\in(0,1]$, $\beta_{j},\bar{\beta }_{j}\in(0,1)$, $\gamma_{j}>1-\frac{1}{p}$, $\bar{\gamma}_{j}>1-\frac{1}{p}$ *such that* $\frac{1}{p}+\alpha_{j}(\beta_{j}-1)+\gamma_{j}-1\ge0$, $\frac {1}{p}+\bar{\alpha}_{j}(\bar{\beta}_{j}-1)+\bar{\gamma}_{j}-1\ge0$, $p(\beta_{j}-1)+1>0$, $p(\bar{\beta}_{j}-1)+1>0$, $p>1$ *for all* $j=1,\ldots,m$.

*If* $u\in C([b_{*}(x_{0})-h,x_{1})\times[y_{0},y_{1}),\mathbb {R}_{+})$ *satisfies the integral inequality*

2.60 $$\begin{aligned}& u^{k}(x,y)\leq c(x,y)+\sum_{j=1}^{M} \int _{b_{j}(x_{0})}^{b_{j}(x)} \int_{c_{j}(y_{0})}^{c_{j}(y)} \bigl(x^{\alpha_{j}}-s^{\alpha_{j}} \bigr)^{\beta_{j}-1}s^{\gamma_{j}-1}\bigl(y^{\bar {\alpha}_{j}}-t^{\bar{\alpha}_{j}} \bigr)^{\bar{\beta}_{j}-1} \\& \hphantom{u^{k}(x,y)\leq{}}{} \times t^{\bar{\gamma}_{j}-1} \hat{g}_{j}(x,y,s,t) \phi_{j}\bigl(u(s,t)\bigr)\hat{\phi}_{j} \Bigl(\max _{\tilde{\eta}\in [s-h, s]}g\bigl(u(\tilde{\eta},t)\bigr) \Bigr) \\& \hphantom{u^{k}(x,y)\leq{}}{} +\sum_{j=M+1}^{m} \int_{b_{j}(x_{0})}^{b_{j}(x)} \int _{c_{j}(y_{0})}^{c_{j}(y)}\hat{g}_{j}(x,y,s,t) \phi_{j}\bigl(u(s,t)\bigr)\hat{\phi }_{j} \Bigl(\max _{\tilde{\eta}\in[s-h, s]}u(\tilde{\eta },t) \Bigr), \\& \hphantom{u^{k}(x,y)\leq{}}{}(x,y)\in[x_{0},x_{1})\times [y_{0}, y_{1}), \\& u(x,y) \leq \hat{ \psi }(x,y), \quad (x,y)\in\bigl[b_{*}(x_{0})-h,x_{0} \bigr]\times [y_{0}, y_{1}), \end{aligned}$$

*where* $\max_{s\in[b_{*}(x_{0})-h,x_{0}]}\hat{\psi}(s,y)\leq( (1+m)^{1-1/q}c(x_{0},y))^{1/k}$ *for all* $y\in[y_{0},y_{1})$.

*Then*

2.61 $$ u(x,y) \leq \biggl(G_{m}^{-1} \bigl(G_{m}\bigl(e_{m}(x,y)\bigr)\bigr) + \int_{b_{m}(x_{0})}^{b_{m}(x)} \int_{c_{m}(y_{0})}^{c_{m}(y)} \tilde{g}_{m}(x,y,s,t)\,dt \,ds\biggr)^{1/(kq)} $$

*for all* $(x,y)\in[x_{0}, X_{2})\times[y_{0},Y_{2})$, *where* $G_{j}^{-1}$*is the inverse of the function*

2.62 $$ G_{j}(u):= \int_{t_{j}}^{t}\frac{ds}{\phi^{q}_{j}(s^{1/(kq)})\hat{\phi }^{q}_{j}(s^{1/(kq)})}, \quad t\ge t_{j}>0, j=1,\ldots,m. $$

*In* (2.61) *and* (2.62), $t_{j}>0$ *is a given constant*, $\frac{1}{p}+\frac{1}{q}=1$, $e_{j}(x,y)$ *is defined recursively by*

2.63 $$\begin{aligned}& \begin{aligned}[b] &e_{1}(x,y)=(1+m)^{q-1}\Bigl( \max_{(\iota,\xi)\in[x_{0}, x ]\times [y_{0},y]}c(\iota,\xi)\Bigr)^{q},\quad \textit{and} \\ &e_{j+1}(x,y):=G_{j}^{-1}\biggl[G_{j} \bigl(e_{j}(x,y)\bigr)+ \int _{b_{j}(x_{0})}^{b_{j}(x)} \int_{c_{j}(y_{0})}^{c_{j}(y)} \tilde{g}_{j}(x,y,s,t) \,dt\,ds\biggr], \\ &\quad j=1,\ldots, m-1, \end{aligned} \end{aligned}$$

2.64 $$\begin{aligned}& \begin{aligned}[b] &\tilde{g}_{j}(x,y,s,t):=(1+m)^{q-1} \bigl({M_{j}} x^{\theta_{j}}{\bar{M}_{j}} y^{\bar{\theta}_{j}}\bigr)^{q/p}\Bigl(\max_{(\iota,\xi)\in[x_{0}, x ]\times[y_{0},y]} \hat{g}_{j}(\iota,\xi,s,t)\Bigr)^{q}, \\ &\quad (x,y)\in [x_{0},x_{1})\times[y_{0},y_{1}), \end{aligned} \end{aligned}$$

2.65 $$\begin{aligned}& \begin{aligned} &M_{j}=\alpha_{j}^{-1}B \biggl(\frac{p(\gamma_{j}-1)+1}{\alpha_{j}}, p(\beta_{j}-1)+1\biggr), \\ &\bar{M}_{j}=\bar{\alpha}_{j}^{-1}B\biggl( \frac{p(\bar{\gamma }_{j}-1)+1}{\bar{\alpha}_{j}}, p(\beta_{j}-1)+1\biggr), \\ &\theta_{j}=p\bigl(\alpha_{j}(\beta_{j}-1)+ \gamma_{j}-1\bigr)+1, \\ &\bar{\theta}_{j}=p\bigl(\bar{\alpha}_{j}(\bar{ \beta}_{j}-1)+\bar{\gamma}_{j}-1\bigr)+1, \quad j=1, \ldots,M \\ &M_{j}=\bar{M}_{j}=1, \qquad \theta_{j}= \bar{\theta}_{j}=1, \quad j=M+1,\ldots,m, \end{aligned} \end{aligned}$$

$X_{2}\in[x_{0}, x_{1})$, $Y_{2}\in[y_{0}, y_{1})$ *are chosen such that*

2.66 $$\begin{aligned}& G_{j}\bigl(r_{j}(X_{2},Y_{2})\bigr)+ \int_{b_{j}(x_{0})}^{b_{j}(X_{1})} \int _{c_{j}(y_{0})}^{c_{j}(Y_{2})} \tilde{g}_{j}(X_{2},Y_{2},s,t) \,dt\,ds \\& \quad \le \int_{t_{j}}^{\infty}\frac{ds}{\phi^{q}_{j}(s^{1/q})\hat{\phi }^{q}_{j}(s^{1/q})} \end{aligned}$$

*for* $j=1,2,\ldots,m$.

### Proof

Let

2.67 $$ \begin{aligned} &\hat{c}(x,y):=\max_{(\tau,\xi)\in[x_{0}, x]\times[y_{0},y] }\bigl\{ a(\tau,\xi)\bigr\} , \quad (x,y)\in[x_{0},x_{1}) \times[y_{0},y_{1}). \\ &\bar{g}_{j}(x,y,s,t) :=\max_{(\iota,\xi)\in[x_{0}, x ]\times[y_{0},y]}g_{j}( \iota ,\xi,s,t), \end{aligned} $$

which are increasing in *x* and *y* and satisfy $\bar {g}_{j}(x,y,s,t)\geq g_{j}(x,y,s,t)\geq0$ for $j=1,2,\ldots,m$. From (2.60), (2.67), and the definition of $\tilde {g}_{j}$, we obtain

2.68 $$\begin{aligned}& u^{k}(x,y) \leq \hat{c}(x,y)+\sum_{j=1}^{M} \int _{b_{j}(x_{0})}^{b_{j}(x)} \int_{c_{j}(y_{0})}^{c_{j}(y)} \bigl(x^{\alpha_{j}}-s^{\alpha_{j}} \bigr)^{\beta_{j}-1}s^{\gamma_{j}-1}\bigl(y^{\bar {\alpha}_{j}}-t^{\bar{\alpha}_{j}} \bigr)^{\bar{\beta}_{j}-1} \\& \hphantom{u^{k}(x,y) \leq{}}{} \times t^{\bar{\gamma}_{j}-1} \bar{g}_{j}(x,y,s,t) \phi_{j}\bigl(u(s,t)\bigr)\hat{\phi}_{j}\Bigl(\max _{\tilde{\eta}\in[s-h, s]}u(\tilde{\eta},t)\Bigr) \\& \hphantom{u^{k}(x,y) \leq{}}{} +\sum_{j=M+1}^{m} \int_{b_{j}(x_{0})}^{b_{j}(x)} \int _{c_{j}(y_{0})}^{c_{j}(y)}\bar{g}_{j}(x,y,s,t) \phi_{j}\bigl(u(s,t)\bigr)\hat{\phi}_{j}\Bigl(\max _{\tilde{\eta}\in[s-h, s]}u(\tilde{\eta},t)\Bigr), \\& \hphantom{u^{k}(x,y) \leq{}}{} (x,y)\in[x_{0},x_{1})\times [y_{0}, y_{1}), \\& u(x,y) \leq \hat{ \psi }(x,y), \quad (x,y)\in\bigl[b_{*}(x_{0})-h,x_{0} \bigr]\times [y_{0}, y_{1}). \end{aligned}$$

Let $\frac{1}{p}+\frac{1}{q}=1$, $p>1$, then $q>0$. By Lemma 1, Hölder’s inequality, (S_4_), and (2.68), we obtain, for all $(x,y)\in\Delta$,

2.69 $$\begin{aligned}& u^{k}(x,y) \\& \quad \leq \hat{c}(x,y)+\sum_{j=1}^{M} \biggl( \int _{b_{j}(x_{0})}^{b_{j}(x)} \int_{c_{j}(y_{0})}^{c_{j}(y)} \bigl(x^{\alpha_{j}}-s^{\alpha_{j}} \bigr)^{p(\beta_{j}-1)}s^{p(\gamma_{j}-1)}\bigl(y^{\bar {\alpha}_{j}}-t^{\bar{\alpha}_{j}} \bigr)^{p(\bar{\beta}_{j}-1)}t^{(\bar{\gamma}_{j}-1)}\,dt\,ds\biggr)^{1/p} \\& \qquad {}\times\biggl( \int _{b_{j}(x_{0})}^{b_{j}(x)} \int_{c_{j}(y_{0})}^{c_{j}(y)}\bar{g}^{q}_{j}(x,y,s,t) \phi^{q}_{j}\bigl(u(s,t)\bigr)\hat{\phi}^{q}_{j} \Bigl(\max_{\tilde{\eta}\in [s-h,s]}u(\tilde{\eta},t)\Bigr) \,dt\,ds\biggr)^{1/q} \\& \qquad {}+\sum_{j=M+1}^{m} \biggl( \int_{b_{j}(x_{0})}^{b_{j}(x)} \int _{c_{j}(y_{0})}^{c_{j}(y)} 1^{p}\,dt\,ds \biggr)^{1/p}\biggl( \int_{b_{j}(x_{0})}^{b_{j}(x)} \int _{c_{j}(y_{0})}^{c_{j}(y)}\bar{g}^{q}_{j}(x,y,s,t) \phi^{q}_{j}\bigl(u(s,t)\bigr) \\& \qquad {}\times\hat{\phi}^{q}_{j}\Bigl(\max_{\tilde{\eta}\in [s-h,s]}u(\tilde{ \eta},t)\Bigr) \,dt\,ds\biggr)^{1/q} \\& \quad \leq \hat{c}(x,y)+\sum_{j=1}^{M} \biggl( \int _{b_{j}(0)}^{x} \int_{0}^{y} \bigl(x^{\alpha_{j}}-s^{\alpha_{j}} \bigr)^{p(\beta_{j}-1)}s^{p(\gamma_{j}-1)}\bigl(y^{\bar {\alpha}_{j}}-t^{\bar{\alpha}_{j}} \bigr)^{p(\bar{\beta}_{j}-1)}t^{(\bar{\gamma}_{j}-1)}\,dt\,ds\biggr)^{1/p} \\& \qquad {} \times\biggl( \int _{b_{j}(x_{0})}^{b_{j}(x)} \int_{c_{j}(y_{0})}^{c_{j}(y)}\bar{g}^{q}_{j}(x,y,s,t) \phi^{q}_{j}\bigl(u(s,t)\bigr)\hat{\phi}^{q}_{j} \Bigl(\max_{\tilde{\eta}\in [s-h,s]}u(\tilde{\eta},t)\Bigr) \,dt\,ds\biggr)^{1/q} \\& \qquad {}+\sum_{j=M+1}^{m} \biggl( \int_{0}^{x} \int_{0)}^{y} 1^{p}\,dt\,ds \biggr)^{1/p}\biggl( \int_{b_{j}(x_{0})}^{b_{j}(x)} \int _{c_{j}(y_{0})}^{c_{j}(y)}\bar{g}^{q}_{j}(x,y,s,t) \phi^{q}_{j}\bigl(u(s,t)\bigr) \\& \qquad {}\times\hat{\phi}^{q}_{j}\Bigl(\max_{\tilde{\eta}\in [s-h,s]}u(\tilde{ \eta},t)\Bigr) \,dt\,ds\biggr)^{1/q} \\& \quad \leq \hat{c}(x,y)+ \sum_{j=1}^{m} \bigl(M_{j}x^{\theta_{j}}\bar{M}_{j}y^{\bar{\theta}_{j}} \bigr)^{1/p}\biggl( \int _{b_{j}(x_{0})}^{b_{j}(x)} \int_{c_{j}(y_{0})}^{c_{j}(y)}\bar{g}^{q}_{j}(x,y,s,t) \\& \qquad {} \times\phi^{q}_{j}\bigl(u(s,t)\bigr) \hat{ \phi}^{q}_{j}\Bigl(\max_{\tilde {\eta}\in[s-h,s]}u(\tilde{ \eta},t)\Bigr) \,dt\,ds\biggr)^{1/q}, \end{aligned}$$

where $0\le b_{j}(t)\le t $, $0\le c_{j}(t)\le t$, $M_{j}$, $\bar {M}_{j}$, $\theta_{j}$, and $\bar{\theta}_{j}$ are given by (2.65) for $j=1,\ldots,m$.

By Jensen’s inequality and (2.69), we get, for all $(x,y)\in \Delta$,

2.70 $$\begin{aligned} u^{kq}(x,y) \leq& (1+m)^{q-1}( \hat{c}^{q}(x,y)+ \sum_{j=1}^{m}\bigl(M_{j}x^{\theta_{j}} \bar{M}_{j}y^{\bar{\theta}_{j}}\bigr)^{q/p} \int _{b_{j}(x_{0})}^{b_{j}(x)} \int_{c_{j}(y_{0})}^{c_{j}(y)}\bar{g}^{q}_{j}(x,y,s,t) \\ &{} \times\phi^{q}_{j}\bigl(u(s,t)\bigr) \hat{ \phi}^{q}_{j}\bigl(\max_{\tilde{\eta }\in[s-h,s]}u(\tilde{\eta},t)\bigr) \,dt\,ds. \end{aligned}$$

By the definition of $e_{1}$ and $\tilde{g}_{j}$, from (2.70) we obtain

2.71 $$\begin{aligned} u^{kq}(x,y) \leq& e_{1}(x,y)+ \sum _{j=1}^{m} \int _{b_{j}(x_{0})}^{b_{j}(x)} \int_{c_{j}(y_{0})}^{c_{j}(y)}\tilde{g}_{j}(x,y,s,t) \\ &{}\times\phi^{q}_{j}\bigl(u(s,t)\bigr) \hat{ \phi}^{q}_{j}\bigl(\max_{\tilde{\eta }\in[s-h,s]}u(\tilde{\eta},t)\bigr) \,dt\,ds,\quad (x,y)\in\Delta. \end{aligned}$$

Concerning (2.71), we consider the auxiliary inequalities

2.72 $$\begin{aligned}& u^{kq}(x,y)\leq e_{1}(X,Y)+ \sum _{j=1}^{m} \int _{b_{j}(x_{0})}^{b_{j}(x)} \int_{c_{j}(y_{0})}^{c_{j}(y)}\tilde{g}_{j}(X,Y,s,t) \\& \hphantom{u^{kq}(x,y)\leq{}}{}\times\phi^{q}_{j}\bigl(u(s,t)\bigr) \hat{\phi}^{q}_{j}\bigl(\max_{\tilde{\eta }\in[s-h,s]}u(\tilde{ \eta},t)\bigr) \,dt\,ds, \quad (x,y)\in[x_{0},X] \times[y_{0},Y], \\& u(x,y)\leq \hat{\psi}(x,y), \quad (x,y)\in \bigl[b_{*}(x_{0})-h,x_{0} \bigr]\times[y_{0},Y], \end{aligned}$$

where $x_{0}\leq X\le X_{2}$ and $y_{0}\leq Y\le Y_{2}$ are chosen arbitrarily.

Since $\max_{s\in[b_{*}(x_{0})-h,x_{0}]}\hat{\psi}(s,y)\leq ((1+m)^{1-1/q}a(x_{0},y))^{\frac{1}{k}}$ for $y\in[y_{0},y_{1})$, $a(x_{0},y)\le\hat{ c}(x_{0},y)$, we have $\max_{s\in[b_{*}(x_{0})-h,x_{0}]}\psi(s,y)\leq (e^{1/q}_{1}(X,Y))^{\frac{1}{k}}$, $y\in[y_{0},Y]$. Define a function $z(x,y): [b_{*}(x_{0})-h, X)\times[y_{0},Y)\rightarrow \mathbb {R}_{+}$ by

$$ z(x,y)= \textstyle\begin{cases} e_{1}(X,Y)+ \sum_{j=1}^{m}\int_{b_{j}(x_{0})}^{b_{j}(x)}\int _{c_{j}(y_{0})}^{c_{j}(y)}\tilde{g}_{j}(X,Y,s,t) \\ \quad {}\times\phi^{q}_{j}(u(s,t))\hat{\phi}^{q}_{j}\bigl(\max_{\tilde{\eta }\in[s-h,s]}u(\tilde{\eta},t)\bigr) \,dt\,ds, \quad (x,y)\in[x_{0},X]\times [y_{0},Y], \\ e_{1}(X,Y), \quad (x,y)\in[b_{*}(x_{0})-h,x_{0}]\times [y_{0}, Y]. \end{cases} $$

Clearly, $z(x,y)$ is increasing in *x*. From (2.72) and the definition of *z*, we have

2.73 $$ u(x,y)\leq z^{1/(kq)}(x,y),\quad (x,y)\in \bigl[b_{*}(x_{0})-h, X\bigr]\times[y_{0},Y]. $$

Then, noting that *z* is increasing, from (2.51) we get for $(s,y)\in[b_{*}(x_{0}), X]\times[y_{0},Y]$

2.74 $$ \max_{\tilde{\eta}\in[s-h, s]} u(\tilde{\eta},y) \leq\max _{\tilde{\eta}\in[s-h, s]} z^{1/(kq)}(\tilde{\eta},y)\le\bigl( \max_{\tilde{\eta}\in[s-h, s]} z(\tilde{\eta},y)\bigr)^{1/(kq)}. $$

From (2.42), (2.73), (2.74), and the definition of *z*, we have

2.75 $$\begin{aligned}& z(x,y)\leq e_{1}(X,Y)+\sum_{j=1}^{m} \int _{b_{j}(x_{0})}^{b_{j}(x)} \int_{b_{j}(y_{0})}^{b_{j}(y)} \tilde{g}_{j}(X,Y,s,t) \phi^{q}_{j}\bigl(z^{1/(kq)}(s,t)\bigr) \\& \hphantom{z(x,y)\leq{}}{}\times\hat{\phi}^{q}_{j} \Bigl(\max _{\tilde{\eta}\in[s-h, s]} \bigl(z^{1/(kq)}(\tilde{\eta},t)\bigr) \Bigr)\,dt\,ds \\& \hphantom{z(x,y)}\leq e_{1}(X,Y)+\sum_{j=1}^{m} \int_{b_{j}(x_{0})}^{b_{j}(x)} \int _{b_{j}(y_{0})}^{b_{j}(y)} \tilde{g}_{j}(X,Y,s,t) \phi^{q}_{j}\bigl(z^{1/(kq)}(s,t)\bigr) \\& \hphantom{z(x,y)\leq{}}{}\times\hat{\phi}^{q}_{j} \Bigl(\bigl(\max _{\tilde{\eta}\in[s-h, s]} z(\tilde{\eta},t)\bigr)^{1/(kq)}\Bigr) \,dt\,ds, \quad (x,y)\in[x_{0}, X] \times[y_{0},Y], \\& z(x,y) \leq e_{1}(X,Y), \quad (x,y)\in\bigl[b_{*}(x_{0})-h,x_{0} \bigr]\times[y_{0}, Y]. \end{aligned}$$

Let $v(t):=t^{1/(kq)}$, which is a continuous and increasing function on $\mathbb {R}_{+}$. Thus $\phi^{q}_{j}(v(t)) $ and $\hat {\phi}^{q}_{j}(v(t))$ ($j=1,\ldots, m$) are continuous and increasing on $\mathbb {R}_{+}$ and positive on $(0,\infty)$. Moreover, since $\phi_{j}\hat{\phi}_{j}\propto\phi_{j+1}\hat{\phi}_{j+1}$, we have $(\phi_{j+1}\circ v)^{q}(\hat{\phi}_{j+1}\circ v)^{q} \propto(\phi _{j}\circ v)^{q}(\hat{\phi}_{j}\circ v)^{q}$ ($j=1,\ldots,m-1$). Taking $g_{j}(x,y,s,t)=\tilde{g}_{j}(X,Y,s,t)$ and $h_{j}(t)=\phi^{q}_{j}(v(t))$, $\bar{h}_{j}(t)=\hat{\phi}^{q}_{j}(v(t))$, $j=1,2,\ldots,m$, in Lemma 2 and (2.75),we obtain

2.76 $$\begin{aligned} z(x,y) \le& G_{m}^{-1}\biggl(G_{m} \bigl(\tilde{e}_{m}(X,Y,x,y)\bigr) \\ &{} + \int_{b_{m}(x_{0})}^{b_{m}(x)} \int_{c_{m}(y_{0})}^{c_{m}(y)} \tilde{ g}_{m}(X,Y,s,t) \,dt\,ds\biggr) \end{aligned}$$

for $x_{0}\le x \le\min\{X, X^{*}_{2}\}$, $y_{0}\le y \le\min\{Y, Y^{*}_{2}\}$, where $\tilde{e}_{j}(X,Y,x,y)$ is defined inductively by $\tilde {e}_{1}(X,Y,x,y):=e_{1}(X,Y)$ and

$$ \tilde{e}_{j}(X,Y,x,y):= G_{j-1}^{-1} \biggl(G_{j-1}\bigl(\tilde {e}_{j-1}(X,Y,x,y)\bigr)+ \int_{b_{j-1}(x_{0})}^{b_{j-1}(x)} \int _{c_{j-1}(y_{0})}^{c_{j-1}(y)} \tilde{g}_{j-1}(X,Y,s,t) \,dt\,ds\biggr) $$

for $j=2,\ldots, m$, and $X^{*}_{2}$, $Y^{*}_{2}$ are chosen such that

2.77 $$\begin{aligned}& G_{j}\bigl(\tilde{e}_{j}(X,Y,\bar{X}_{1}, \bar{Y}_{1})\bigr)+ \int _{b_{j}(x_{0})}^{b_{j}(X_{2})} \int_{c_{j}(y_{0})}^{c_{j}(\bar{Y}_{1})} \tilde{g}_{j}(X,Y,s,t) \\& \quad \le \int_{t_{j}}^{\infty}\frac{ds}{\tilde{\omega}^{q}_{j}(\varphi ^{-1}(s^{1/q}))} \end{aligned}$$

for $j=1,\ldots,m$.

Note that $X^{*}_{2}=X_{2}$ and $Y^{*}_{2}=Y_{2}$. It follows from (2.73) and (2.76) that

2.78 $$\begin{aligned} u(x,y) \le& \biggl(G_{m}^{-1}\biggl(G_{n}\bigl( \tilde{g}_{m}(X,Y,x,y)\bigr) \\ &{}+ \int_{b_{m}(x_{0})}^{b_{m}(x)} \int_{c_{m}(y_{0})}^{c_{m}(y)} \tilde{ g}_{m}(X,Y,s,t) \,dt\,ds\biggr)\biggr)^{1/(kq)} \end{aligned}$$

for $x_{0}\le x \le\min\{X, X^{*}_{2}\}$, $y_{0}\le y \le\min\{Y, Y^{*}_{2}\}$.

Taking $x=X$, $y=Y$ in (2.56), we have

2.79 $$\begin{aligned} u(X,Y) \le& \biggl(G_{m}^{-1}\biggl(G_{m}\bigl( \tilde{e}_{m}(X,Y,X,Y)\bigr) \\ &{}+ \int_{b_{m}(x_{0})}^{b_{m}(X)} \int_{c_{m}(y_{0})}^{c_{m}(Y)} \tilde{ g}_{m}(X,Y,s,t) \,dt\,ds\biggr)\biggr)^{1/(kq)} \end{aligned}$$

for $x_{0}\le X\le X_{2}$, $y_{0}\le Y\le Y_{2}$. It is easy to verify $\tilde {e}_{m}(X,Y,X,Y)= e_{m}(X,Y)$. Thus, (2.57) can be written as

2.80 $$\begin{aligned} u(X,Y) \le& \biggl(G_{m}^{-1}\biggl(G_{n} \bigl(r_{m}(X,Y)\bigr) \\ &{}+ \int_{b_{m}(x_{0})}^{b_{m}(X)} \int_{c_{m}(y_{0})}^{c_{m}(Y)} \tilde{ g}_{m}(X,Y,s,t) \,dt\,ds\biggr)\biggr)^{1/(kq)}. \end{aligned}$$

Since $X,Y$ are arbitrary, replacing *X* and *Y* with *x* and *y*, respectively, we get

2.81 $$\begin{aligned} u(x,y) \le& \biggl(G_{m}^{-1}\biggl(G_{n} \bigl(e_{m}(x,y)\bigr) \\ &{}+ \int_{b_{m}(x_{0})}^{b_{m}(x)} \int_{c_{m}(y_{0})}^{c_{m}(y)} \tilde{ g}_{m}(x,y,s,t) \,dt\,ds\biggr)\biggr)^{1/(kq)} \end{aligned}$$

for all $(x,y)\in[x_{0}, X_{2}]\times[y_{0},Y_{2}]$. This completes the proof. □

### Corollary 2.3

*Let the following conditions be fulfilled*:

(B_1_): *all* $b_{j}\in C^{1}([x_{0},x_{1}),\mathbb {R}_{+})$ *and* $c_{j}\in C^{1}([y_{0},y_{1}), [y_{0},y_{1}))$ *are nondecreasing with* $b_{j}(x)\leq x$ *on* $[x_{0},x_{1})$, $c_{j}(y)\le y$ *on* $[y_{0},y_{1})$, *and* $c_{j}(y_{0})=y_{0}$ *for all* $j=1,\ldots,m$;
(B_2_): $a\in C(\Delta, \mathbb{R}_{+})$ *and* $\hat{\psi}\in C(\Xi,\mathbb {R}_{+})$, $\varphi_{1} \in C(\mathbb {R}_{+},\mathbb {R}_{+})$, *and* $\varphi_{1}$ *is strictly increasing such that* $\lim_{t\rightarrow\infty}\varphi(t)=\infty$,*and* $f_{j}\in C(\Delta\times[b_{*}(x_{0}),x_{1})\times[y_{0},y_{1}),\mathbb{R}_{+})$ *for all* $j=1,\ldots, m$;
(B_3_): *all* $\psi_{j}$ ($j=1,\ldots,m$) *are continuous and increasing functions on* $\mathbb {R}_{+}$ *and positive on* $(0,+\infty)$ *such that* $\psi_{1}\propto\psi_{2}\propto\ldots\propto\psi_{m}$;
(B_4_): $\alpha_{j}, \bar{\alpha}_{j}\in(0,1]$, $\beta_{j},\bar{\beta}_{j}\in(0,1)$, $\gamma_{j}>1-\frac{1}{p}$, $\bar{\gamma}_{j}>1-\frac{1}{p}$ *such that* $\frac{1}{p}+\alpha_{j}(\beta_{j}-1)+\gamma_{j}-1\ge0$, $\frac {1}{p}+\bar{\alpha}_{j}(\bar{\beta}_{j}-1)+\bar{\gamma}_{j}-1\ge0$, $p(\beta_{j}-1)+1>0$, $p(\bar{\beta}_{j}-1)+1>0$, $p>1$, $j=1,2,\ldots,m$;
(B_5_): $u\in C([b_{*}(x_{0})-h,x_{1})\times[y_{0},y_{1}),\mathbb {R}_{+})$ *satisfies the integral inequality*
  2.82 $$ \begin{aligned} &\varphi_{1}\bigl(u(x,y) \bigr) \leq a(x,y)+\sum_{j=1}^{M} \int _{b_{j}(x_{0})}^{b_{j}(x)} \int_{c_{j}(y_{0})}^{c_{j}(y)} \bigl(x^{\alpha_{j}}-s^{\alpha_{j}} \bigr)^{\beta_{j}-1}s^{\gamma_{j}-1}\bigl(y^{\bar {\alpha}_{j}}-t^{\bar{\alpha}_{j}} \bigr)^{\bar{\beta}_{j}-1} \\ &\hphantom{\varphi_{1}(u(x,y)) \leq{}}{} \times t^{\bar{\gamma}_{j}-1} f_{j}(x,y,s,t) \psi_{j}\bigl(u(s,t)\bigr)\,dt\,ds \\ &\hphantom{\varphi_{1}(u(x,y)) \leq{}}{} +\sum_{j=M+1}^{m} \int_{b_{j}(x_{0})}^{b_{j}(x)} \int _{c_{j}(y_{0})}^{c_{j}(y)}\bigl(x^{\alpha_{j}}-s^{\alpha_{j}} \bigr)^{\beta_{j}-1}s^{\gamma_{j}-1} \bigl(y^{\bar{\alpha}_{j}}-t^{\bar{\alpha}_{j}} \bigr)^{\bar{\beta}_{j}-1} \\ &\hphantom{\varphi_{1}(u(x,y)) \leq{}}{} \times t^{\bar{\gamma}_{j}-1}f_{j}(x,y,s,t) \psi_{j} \Bigl(\max_{\tilde {\eta}\in[s-h,s]}u(\tilde{\eta},t) \Bigr) \,dt\,ds, \\ &\hphantom{\varphi_{1}(u(x,y)) \leq{}}{} (x,y)\in[x_{0},x_{1})\times [y_{0}, y_{1}), \\ &u(x,y) \leq \hat{\psi }(x,y), \quad (x,y)\in\bigl[b_{*}(x_{0})-h,x_{0} \bigr]\times [y_{0}, y_{1}), \end{aligned} $$
  *where* $\max_{s\in[b_{*}(x_{0})-h,x_{0}]}\hat{\psi}(s,y)\leq \varphi_{1}^{-1}( (1+m)^{1-1/q}a(x_{0},y))$ *for all* $y\in[y_{0},y_{1})$.

*Then*

2.83 $$\begin{aligned} u(x,y) \leq& \varphi_{1}^{-1}\biggl(\check{G}_{m}^{-1} \biggl(\check{G}_{m}\bigl(r_{m}(x,y)\bigr) \\ &{} + \int_{b_{m}(x_{0})}^{b_{m}(x)} \int_{c_{m}(y_{0})}^{c_{m}(y)} \tilde{f}_{m}(x,y,s,t)\,dt \,ds\biggr)^{1/q}\biggr) \end{aligned}$$

*for all* $(x,y)\in[x_{0}, X_{2})\times[y_{0},Y_{2})$, *where* $G_{j}^{-1}$ *is the inverse of the function*

2.84 $$ \check{G}_{j}(t):= \int_{t_{j}}^{t}\frac{ds}{\psi^{q}_{j}(\varphi _{1}^{-1}(s^{1/q}))},\quad t\ge t_{j}>0, j=1,2,\ldots,m, $$

$t_{j}$ *is a given constant*, $r_{j}(x,y)$ *is defined recursively by*

2.85 $$\begin{aligned}& r_{1}(x,y)=(1+m)^{q-1}\Bigl(\max_{(\iota,\xi)\in[x_{0}, x ]\times [y_{0},y]}a( \iota,\xi)\Bigr)^{q},\quad \textit{and} \\& r_{j+1}(x,y):= \check{G}_{j}^{-1}\biggl[ \check{G}_{j}\bigl(r_{j}(x,y)\bigr)+ \int _{b_{j}(x_{0})}^{b_{j}(x)} \int_{c_{j}(y_{0})}^{c_{j}(y)} \tilde{f}_{j}(x,y,s,t) \,dt\,ds\biggr], \\& \quad j=1,\ldots, m-1, \end{aligned}$$

2.86 $$\begin{aligned}& \begin{aligned}[b] &\tilde{f}_{j}(x,y,s,t):=(1+m)^{q-1} \bigl({M_{j}} x^{\theta_{j}}{\bar{M}_{j}} y^{\bar{\theta}_{j}}\bigr)^{q/p}\Bigl(\max_{(\iota,\xi)\in[x_{0}, x ]\times[y_{0},y]} \check{f}_{j}(\iota,\xi,s,t)\Bigr)^{q}, \\ &\quad (x,y)\in [x_{0},x_{1})\times[y_{0},y_{1}), \end{aligned} \end{aligned}$$

$M_{j}:=\alpha_{j}^{-1}B(\frac{p(\gamma_{j}-1)+1}{\alpha_{j}}, p(\beta_{j}-1)+1)$, $\bar{M}_{j}:=\bar{\alpha}_{j}^{-1}B(\frac{p(\bar {\gamma}_{j}-1)+1}{\bar{\alpha}_{j}}, p(\beta_{j}-1)+1)$, $\theta_{j}:=p(\alpha_{j}(\beta_{j}-1)+\gamma _{j}-1)+1$, $\bar{\theta}_{j}:=p(\bar{\alpha}_{j}(\bar{\beta }_{j}-1)+\bar{\gamma}_{j}-1)+1$, $\frac{1}{p}+\frac{1}{q}=1$, $X_{2}\in[x_{0}, x_{1})$, $Y_{2}\in[y_{0}, y_{1})$ *are chosen such that*

2.87 $$ \check{G}_{j}\bigl(r_{j}(X_{2},Y_{2}) \bigr)+ \int_{b_{j}(x_{0})}^{b_{j}(X_{1})} \int _{c_{j}(y_{0})}^{c_{i-1}(Y_{2})} \tilde{f}_{j}(X_{2},Y_{2},s,t) \,dt\,ds\le \int_{t_{j}}^{\infty}\frac {ds}{\tilde{\omega}^{q}_{j}(\varphi^{-1}(s^{1/q}))} $$

*for* $j=1,2,\ldots,m$.

### Proof

Applying Theorem 2.1 to specified $\omega_{j}(u)\equiv\psi _{j}(u)$ ($j=1,\ldots,M$), $\mu_{j}(u)\equiv1$ ($j=1,\ldots,M$), $\omega_{j}(u)\equiv1$ ($j=M+1,\ldots,m$), $\mu_{j}(u)\equiv\psi_{j}(u)$ ($j=M+1,\ldots,m$), $f_{j}(x,y,s,t)=\check{f}_{j}(x,y,s,t)$, $g(t)=t$, from (2.82) we obtain estimate (2.83). The proof is complete. □

## Applications

Consider a nonlinear weakly singular integral equation with maxima

3.1 $$ \textstyle\begin{cases} z(x,y)=a(x,y)+\int_{x_{0}}^{x}\int_{y_{0}}^{y}(x-s)^{\theta _{1}-1}s^{\gamma_{1}-1}(y-t)^{\theta_{2}-1}t^{\gamma_{2}-1} \\ \hphantom{z(x,y)={}}{}\times F(x,y, s,t,z(s,t),\max_{ \tilde{\eta}\in [s-h,s]}z( \tilde{\eta},t))\, ds\, dt, \quad (x,y)\in\Delta, \\ z(x,y)=\psi(x,y), \quad (x,y)\in[x_{0}-h,x_{0}]\times[y_{0}, y_{1}), \end{cases} $$

where $F\in C(\Delta\times\mathbb {R}^{4},\mathbb {R})$, *h* is a positive constant, $\psi\in C([x_{0}-h,x_{0}]\times[y_{0},y_{1}),\mathbb{R})$, $a\in C(\Delta, \mathbb {R})$, $\theta_{j}\in(0,1)$, and $p(\gamma_{j}-1)+1>0$ such that $\frac{1}{p}+\theta_{j}+\gamma_{j}-2\ge0$ and $p(\theta _{j}-1)+1>0$, $p>1$, $j=1,2$.

The following result gives an estimate for its solutions.

### Corollary 3.1

*Suppose that functions* *F* *in* (3.1) *satisfy*

3.2 $$ \bigl\vert F(x,y,s,t,u,v) \bigr\vert \le h_{1}(x,y,s,t) \mu_{1}\bigl( \vert u \vert \bigr)+h_{2}(x,y,s,t) \mu_{2}\bigl( \vert v \vert \bigr), $$

*where* $h_{j}\in C([x_{0},x_{1})\times[y_{0},y_{1})\times\mathbb {R}^{2},\mathbb {R}_{+})$, *and* $h_{j}(x,y,s,t)$ *is nondecreasing in* *x* *and* *y* *for each fixed* *s* *and* *t*, *and* $\mu_{j}\in C(\mathbb {R}_{+},(0,\infty))$ ($j=1,2$) *such that* $\mu_{1}\propto\mu_{2}$, $\max_{s\in[x_{0}-h,x_{0}]}\psi(s,y)\le3^{1-1/q}|a(x_{0}, y)|$ *for all* $y\in[y_{0}, y_{1})$.

*Then any solution* $z(x,y)$ *of* (3.1) *has the estimate*

3.3 $$\begin{aligned}& \bigl\vert z(x,y) \bigr\vert \\& \quad \le \biggl[ {Q_{2}}^{-1} \biggl(Q_{2}\bigl(\gamma(x,y)\bigr)+3^{q-1} \bigl(M_{1}x^{\delta _{1}}M_{2}y^{\delta_{2}} \bigr)^{q/p} \int_{x_{0}}^{x} \int_{y_{0}}^{y}h_{2}(x,y,s,t)dt \,ds \biggr) \biggr]^{1/q} \end{aligned}$$

*for all* $(x,y)\in[x_{0},X_{1})\times[y_{0},Y_{1})$, *where*

$$\begin{aligned}& \gamma(x,y) := Q_{1}^{-1} \biggl(Q_{1}\bigl( \eta _{1}(x,y)\bigr)+3^{q-1}\bigl(M_{1}x^{\delta_{1}}M_{2}y^{\delta_{2}} \bigr)^{q/p} \int _{x_{0}}^{x} \int_{y_{0}}^{y}h^{q}_{1}(x,y,s,t) \,dt\,ds \biggr), \\ & \eta_{1}(x,y) := 3^{q-1}\Bigl(\max_{(s,t)\in [x_{0},x]\times[y_{0},y]} \bigl\vert a(s,t) \bigr\vert \Bigr)^{q},\qquad Q_{1}(u):= \int_{u_{1}}^{u}\frac{ds}{\mu_{1}^{q}(s^{\frac{1}{q}})}, \quad u\ge u_{1}>0, \\ & Q_{2}(u) := \int_{u_{1}}^{u}\frac{ds}{\mu_{2}^{q}(s^{\frac{1}{q}})},\quad u\ge u_{2}>0, \end{aligned}$$

$M_{j}:=B(p(\gamma_{j}-1)+1, p(\theta_{j}-1)+1)$ ($j=1,2$), $\delta _{j}:=p(\theta_{j}+\gamma_{j}-2)+1$, $j=1,2$, $\frac{1}{p}+\frac{1}{q}=1$, *and constants* $u_{1}$, $u_{2}$ *are given arbitrarily*, $X_{1}\in[x_{0}, x_{1})$, $Y_{1}\in[y_{0}, y_{1})$ *are chosen such that*

$$\begin{aligned}& Q_{1}\bigl(\gamma_{1}(X_{1},Y_{1}) \bigr)+3^{q-1}\bigl(M_{1}X_{1}^{\delta_{1}}M_{2}Y_{1}^{\delta _{2}} \bigr)^{q/p} \int_{x_{0}}^{X_{1}} \int_{y_{0}}^{Y_{1}}h^{q}_{1}(X_{1},Y_{1},s,t) \,dt\,ds \le \int_{u_{1}}^{\infty}\frac{ds}{\mu_{1}^{q}(s^{\frac{1}{q}})}, \\& Q_{2}\bigl(\gamma_{2}(X_{1},Y_{1}) \bigr)+3^{q-1}\bigl(M_{1}X_{1}^{\delta_{1}}M_{2}Y_{1}^{\delta _{2}} \bigr)^{q/p} \int_{x_{0}}^{x} \int_{y_{0}}^{y}h^{q}_{2}(X_{1},Y_{1},s,t) \,dt\,ds \le \int_{u_{2}}^{\infty}\frac{ds}{\mu_{2}^{q}(s^{\frac{1}{q}})}. \end{aligned}$$

### Proof

From (3.1) we obtain

3.4 $$ \begin{aligned} & \bigl\vert z(x,y) \bigr\vert \le \bigl\vert a(x,y) \bigr\vert + \int_{x_{0}}^{x} \int _{y_{0}}^{y}(x-s)^{\theta_{1}-1}s^{\gamma_{1}-1}(y-t)^{\theta _{2}-1}t^{\gamma_{2}-1} \\ &\hphantom{ \bigl\vert z(x,y) \bigr\vert \le{}}{}\cdot \Bigl\vert F\Bigl(x,y, s,t,z(s,t),\max _{ \tilde{\eta}\in[s-h,s]}z( \tilde{\eta},t)\Bigr) \Bigr\vert \,dt\,ds \\ &\hphantom{ \bigl\vert z(x,y) \bigr\vert }\le \bigl\vert a(x,y) \bigr\vert + \int_{x_{0}}^{x} \int_{y_{0}}^{y}(x-s)^{\theta _{1}-1}s^{\gamma_{1}-1}(y-t)^{\theta_{2}-1}t^{\gamma_{2}-1} \\ &\hphantom{ \bigl\vert z(x,y) \bigr\vert \le{}}{}\cdot h_{1}(x,y,s,t) \mu _{1}\bigl( \bigl\vert z(s,t) \bigr\vert \bigr)\,dt\,ds \\ &\hphantom{ \bigl\vert z(x,y) \bigr\vert \le{}}{}+ \int_{x_{0}}^{x} \int_{y_{0}}^{y}(x-s)^{\theta_{1}-1}s^{\gamma _{1}-1}(y-t)^{\theta_{2}-1}t^{\gamma_{2}-1} h_{2}(x,y,s,t) \\ &\hphantom{ \bigl\vert z(x,y) \bigr\vert \le{}}{}\cdot\mu_{2}\Bigl( \Bigl\vert \max _{ \tilde{\eta}\in[s-h,s]}z( \tilde {\eta},t)\Bigr) \Bigr\vert )\,dt\,ds,\quad (x,y)\in\Delta, \\ & \bigl\vert z(x,y) \bigr\vert \le \bigl\vert \psi(x,y) \bigr\vert , \quad (x,y)\in [x_{0}-h,x_{0}]\times[y_{0},y_{1}). \end{aligned} $$

Set $v(x,y)=|z(x,y)|$ for all $(x,y)\in[x_{0}-h,x_{1})\times[y_{0},y_{1})$. From (3.4) we get

3.5 $$\begin{aligned}& v(x,y) \le \bigl\vert a(x,y) \bigr\vert + \int_{x_{0}}^{x} \int _{y_{0}}^{y}(x-s)^{\theta_{1}-1}s^{\gamma_{1}-1}(y-t)^{\theta_{2}-1}t^{\gamma _{2}-1} \\& \hphantom{v(x,y) \le{}}{} \cdot h_{1}(x,y,s,t) \mu_{1}\bigl(v(s,t)\bigr)\,dt\,ds \\& \hphantom{v(x,y) \le{}}{}+ \int_{x_{0}}^{x} \int_{y_{0}}^{y}(x-s)^{\theta_{1}-1}s^{\gamma _{1}-1}(y-t)^{\theta_{2}-1}t^{\gamma_{2}-1} h_{2}(x,y,s,t) \\& \hphantom{v(x,y) \le{}}{}\cdot\mu_{2}\Bigl(\max_{ \tilde{\eta}\in[s-h,s]} \bigl\vert v( \tilde {\eta},t) \bigr\vert \Bigr)\,dt\,ds,\quad (x,y)\in\Delta, \\& v(x,y) \le \bigl\vert \psi(x,y) \bigr\vert ,\quad (x,y) \in[x_{0}-h,x_{0}]\times[y_{0},y_{1}). \end{aligned}$$

Applying Corollary 2.3 to the specified $M=1$, $m=2$, $\varphi_{1} (u)=u$, $f_{j}(x,y,s,t)=h_{j}(x,y,s,t)$, $b_{j}(t)=t$, $c_{j}(t)=t$, $\alpha_{j}=\bar{\alpha}_{j}=1$, $g(t)=t$, we obtain (3.3) from (3.5). □

### Corollary 3.2

*Suppose that functions* *F* *and* *ψ* *in* (3.1) *satisfy*

3.6 $$ \bigl\vert F(x,y,s_{1},t_{1})-F(x,y,s_{2},t_{2}) \bigr\vert \leq h_{1}(x,y) \vert s_{1}-s_{2} \vert +h_{2}(x,y) \vert t_{1}-t_{2} \vert $$

*for all* $(x,y)\in\Delta$ *and* $s_{j},t_{j}\in\mathbb {R}$ ($i =1,2$), *where* $h_{j}\in C(\Delta,\mathbb {R}_{+})$. *Then system* (3.1) *has at most one solution on* Δ.

### Proof

Assume that equation (3.1) has two solutions $u(x,y)$, $v(x,y)$. By the equivalent integral equation (3.1), we have

3.7 $$\begin{aligned} \bigl\vert u(x,y)-v(x,y) \bigr\vert \le& \int_{x_{0}}^{x} \int _{y_{0}}^{y}(x-s)^{\theta_{1}-1}s^{\gamma_{1}-1}(y-t)^{\theta_{2}-1}t^{\gamma _{2}-1} h_{1}(s,t) \bigl\vert u(s,t)-v(s,t) \bigr\vert \,dt\,ds \\ &{}+ \int_{x_{0}}^{x} \int_{y_{0}}^{y} (x-s)^{\theta _{1}-1}s^{\gamma_{1}-1}(y-t)^{\theta_{2}-1}t^{\gamma_{2}-1}h_{2}(s,t) \\ &{} \cdot \Bigl\vert \max_{ \tilde{\eta}\in[s-h,s]}u( \tilde{\eta},t)-\max _{ \tilde{\eta}\in[s-h,s]}v( \tilde{\eta},t) \Bigr\vert \,dt\,ds \end{aligned}$$

for all $(x,y)\in[x_{0},x_{1})\times[y_{0},y_{1})$. Since $u(x, y)$ is a continuous function, it implies that, for any fixed $t \in[y_{0}, y]$ and $s \in[x_{0}, x]$, there exists $\tau\in [s-h, s]$ such that $\max_{ \tilde{\eta}\in[s-h,s]}u( \tilde{\eta },t) = u(\tau,t)$ holds. Now we suppose $\max_{ \tilde{\eta }\in[s-h,s]}u( \tilde{\eta},t)\ge\max_{ \tilde{\eta}\in [s-h,s]}v( \tilde{\eta},t)$ and have

3.8 $$\begin{aligned} \Bigl\vert \max_{ \tilde{\eta}\in[s-h,s]}u( \tilde{\eta},t)-\max _{ \tilde{\eta}\in[s-h,s]}v( \tilde{\eta},t) \Bigr\vert =& \Bigl\vert u( \tau,t)-\max_{ \tilde{\eta}\in[s-h,s]}v( \tilde{\eta},t) \Bigr\vert \\ \le& \bigl\vert u(\tau,t)-v(\tau,t) \bigr\vert \le\max_{ \tilde{\eta}\in [s-h,s]} \bigl\vert u( \tilde{\eta},t)-v( \tilde{\eta},t) \bigr\vert . \end{aligned}$$

It follows from (3.7) and (3.8) that

3.9 $$\begin{aligned} \bigl\vert u(x,y)-v(x,y) \bigr\vert \le& \int_{x_{0}}^{x} \int_{y_{0}}^{y} (x-s)^{\theta _{1}-1}s^{\gamma_{1}-1}(y-t)^{\theta_{2}-1}t^{\gamma _{2}-1}h_{1}(s,t) \bigl\vert u(s,t)-v(s,t) \bigr\vert \,dt\,ds \\ &{}+ \int_{x_{0}}^{x} \int_{y_{0}}^{y} (x-s)^{\theta _{1}-1}s^{\gamma_{1}-1}(y-t)^{\theta_{2}-1}t^{\gamma_{2}-1}h_{2}(s,t) \\ &{} \cdot\max_{ \tilde{\eta}\in[s-h,s]} \bigl\vert u( \tilde{\eta },t)-v( \tilde{\eta},t) \bigr\vert \,dt\,ds. \end{aligned}$$

Let

$$\phi(x,y):= \bigl\vert u(x,y)-v(x,y) \bigr\vert , \quad (x,y)\in\bigl[ \alpha(x_{0})-h, x_{0}\bigr]\times [y_{0}, y_{1}). $$

From (3.7) we obtain

3.10 $$\begin{aligned}& \phi(x,y) \le \int_{x_{0}}^{x} \int_{y_{0}}^{y} (x-s)^{\theta _{1}-1}s^{\gamma_{1}-1}(y-t)^{\theta_{2}-1}t^{\gamma_{2}-1}h_{1}(s,t) \phi(s,t)\,dt\,ds \\& \hphantom{\phi(x,y) \le{}}{} + \int_{x_{0}}^{x} \int_{y_{0}}^{y} (x-s)^{\theta_{1}-1}s^{\gamma _{1}-1}(y-t)^{\theta_{2}-1}t^{\gamma_{2}-1}(x-s)^{\theta_{1}-1}s^{\gamma_{1}-1} \\& \hphantom{\phi(x,y) \le{}}{} \cdot(y-t)^{\theta_{2}-1}t^{\gamma_{2}-1}h_{2}(s,t) \max_{ \tilde{\eta}\in[s-h,s]}\phi( \tilde{\eta},t)\, dt \, d\eta, \\& \hphantom{\phi(x,y) \le{}}{}(x,y)\in[x_{0},x_{1}) \times[y_{0},y_{1}), \\& \phi(x,y) \le 0, \quad (x,y)\in [x_{0}-h,x_{0}] \times[y_{0},y_{1}). \end{aligned}$$

Let $\varepsilon>0$ be an arbitrary number. Then from (3.10) we have

3.11 $$ \begin{aligned} &\phi(x,y)\le \varepsilon+ \int_{x_{0}}^{x} \int_{y_{0}}^{y} (x-s)^{\theta_{1}-1}s^{\gamma_{1}-1}(y-t)^{\theta_{2}-1}t^{\gamma _{2}-1}h_{1}(s,t) \phi(s,t)\,dt\,ds \\ &\hphantom{\phi(x,y)\le{}}{}+ \int_{x_{0}}^{x} \int_{y_{0}}^{y} (x-s)^{\theta_{1}-1}s^{\gamma _{1}-1}(y-t)^{\theta_{2}-1}t^{\gamma_{2}-1} \\ &\hphantom{\phi(x,y)\le{}}{} \cdot h_{2}(s,t) \max_{ \tilde{\eta}\in[\alpha(s)-h,\alpha(s)]}\phi( \tilde{\eta},t)\, dt\, d\eta, \\ &\hphantom{\phi(x,y)\le{}}{} (x,y)\in[x_{0},x_{1}) \times[y_{0},y_{1}), \\ &\phi(x,y)\le 0, \quad (x,y)\in [x_{0}-h,x_{0}] \times[y_{0},y_{1}). \end{aligned} $$

Applying Corollary 2.3 to specified $N=1$, $m=2$, $\varphi _{1}(u)=u$, $g(t)=t$, $b_{j}(t)=c_{j}(t)=t$, $f_{j}(x,y,s,t)=h_{2}(s,t)$, $j=12$, $a(x,y)=\epsilon$, from (3.11) we obtain, for all $(x,y)\in\Delta$,

3.12 $$\begin{aligned}& \phi(x,y) \\& \quad \leq 3^{\frac{q-1}{q}}\varepsilon\exp \biggl(q^{-1}\biggl(3^{\frac {q-1}{q}}\bigl(M_{1}x^{\delta_{1}} \bar{M}_{1}y^{\delta_{2}}\bigr)^{\frac{q}{p}} \int_{x_{0}}^{x} \int_{y_{0}}^{y}\bigl(h_{1}^{q}(s,t)+h_{2}^{q}(s,t) \bigr)\,dt\,ds\biggr)\biggr), \end{aligned}$$

where $\frac{1}{p}+\frac{1}{q}=1$, $M_{j}$ and $\delta_{j}$ ($j=1,2$) are defined as in Corollary 3.1. Letting $\varepsilon\rightarrow 0$, we obtain the uniqueness of the solution of equation (3.1). The uniqueness is proved. □
